# Elevated hexosylceramides in Parkinson’s disease cause gene upregulations in neurons mimicking responses to pathogens

**DOI:** 10.1038/s41531-025-01114-9

**Published:** 2025-08-30

**Authors:** Luisa Franck, Lisa Hahnefeld, Lucie Valek, Katharina Klatt-Schreiner, Annett Wilken-Schmitz, Mohamad Wessam Alnouri, Sandra Trautmann, Marc-Philipp Weyer, Dominique Thomas, Robert Gurke, Stefan Offermanns, Gerd Geisslinger, Irmgard Tegeder

**Affiliations:** 1 Goethe University Frankfurt, Faculty of Medicine, Institute of Clinical Pharmacology, Frankfurt am Main, Germany; 2 Fraunhofer Institute for Translational Medicine and Pharmacology ITMP and Fraunhofer Cluster of Excellence for Immune Mediated Diseases CIMD, Frankfurt am Main, Germany; 3 Max Planck Institute for Heart and Lung Research, Department of Pharmacology, Bad Nauheim, Germany

**Keywords:** Parkinson's disease, High-throughput screening, Transgenic organisms, Metabolomics, Next-generation sequencing, Microscopy, Diagnostic markers

## Abstract

Parkinson’s Disease (PD) is driven by pathological aggregates of alpha-synuclein (αSyn), whose formation is facilitated by impaired glycosphingolipid metabolism via acidic glucocerebrosidase (GCase). We investigated glucosylceramide (GlcCer) accumulation in human, mouse, and cellular PD models. Lipidomic analyses revealed elevated plasma GlcCer, especially GlcCer24:1, and a shift in phosphatidylcholine (PC) species in PD patients. PD patient skin fibroblasts accumulated more GlcCer under lysosomal stress. GlcCer and sulfatides (SHexCer) were increased in Pink1^−/−^SNCA^A53T^ PD mouse brains, and HT22 neurons exposed to preformed αSyn fibrils accumulated GlcCer and ceramides. GlcCer24:1 enhanced fibril toxicity, but had no direct or indirect effect on G-protein coupled receptors. RNAseq of GlcCer24:1-treated dorsal root ganglion neurons showed upregulation of glycolipid response genes, similar to pathogen-related signaling. These data indicate extracellular GlcCer is elevated in PD and triggers innate immune responses in sensory neurons.

## Introduction

The pathology of PD is caused by deposits of oligomeric forms of alpha-synuclein (gene *SNCA*, αSyn)^1,2^. Formation of αSyn fibrils is precipitated by glucosylceramides or their glucosylsphingosine metabolites^3–5^, which per se can form twisted ribbon, amyloid-like structures^6^. Oligomers and fibrils of αSyn spread from cell to cell via extracellular vesicles^7^ or directly via tunneling nanotubes^8^ or prion-like cell-to-cell transfer^9^. The primary and probably only pathway for removal is via autophagolysosomal degradation^10–13^. If it fails and lysosomes are overloaded with αSyn^14^, or if the lysosomal pH and calcium gradients decline under the load of accumulated lipids, lysosomal membranes become leaky^15^. The resulting release of proteases into the cytoplasm leads to autoproteolysis if it is not prevented by lysophagy, i.e. autophagy of damaged lysosomes^15,16^. Lysophagy can prevent cell death to some extent but eventually leads into a vicious cycle unless overloaded or damaged lysosomes are expelled as exosomes for temporary relief^17^, but it results in further spreading of αSyn^18,19^. Accumulation of αSyn in lysosomes interferes with the catalytic activity of lysosomal enzymes, in particular glucocerebrosidase 1 (gene *GBA1*, GCase), which is required for degradation of glucosylceramides (GlcCer). In turn, GCase malfunctioning exacerbates deficits of αSyn-degradation^3,20–22^. Accordingly, mutations in genes involved in glycosphingolipid transport such as *ATP10B*^23^ or degradation such as *VPS35*^24,25^ increase the risk and severity of PD. Particularly mutant GBA1 accelerates αSyn pathology^26,27^. It is still not completely understood how heterozygous PD-associated *GBA1* mutations lead to a strong increase of the risk for developing PD^28,29^ and the severity of PD, in particular PD-associated dementia. Homozygous “loss-of-function” *GBA1* mutations are causative for Gaucher disease^30^. Such *GBA1* mutations, but not the most frequent PD-associated E326K mutation, lead to protein misfolding and hence early degradation, or failure of transport and delivery to the lysosomes^31^, which depends on the shuttle protein, lysosome integrated membrane protein 2 (LIMP2)^32,33^. However, misfolding or substantial loss of GCase protein expression or loss of catalytic activity is mostly not seen in heterozygous carriers (as in PD), and mechanistically, the bidirectional pathology of dysfunctional GCase with αSyn is only partially understood^34^. It was hypothesized that mutant GCase directly precipitates αSyn oligomerization, that accumulation of GCase substrates, mostly GlcCer and other glycosphingolipids, drive αSyn oligomerization, that GCase deficiency results in a loss of lysosomal function and capacity for clearance and turnover of αSyn, that increased oligomeric αSyn in the lysosomes impairs membrane targeting and hence, activity of GCase, and that GCase substrates in turn shield αSyn oligomers from degradation^21,35^, all finally leading to lysosomal leakage and impairment of mitochondria^36^. High levels of mutant misfolded GCase protein may also evoke an excessive unfolded-protein-response in the endoplasmic reticulum^37^ or saturation of the ubiquitin–proteasome pathway^38^, and defective generation of extracellular vesicles^39^. It is likely that multiple mechanisms are involved in the bidirectional GCase-αSyn pathology in PD. Dopaminergic neurons of the substantia nigra are particularly vulnerable to αSyn oligomers, and their death manifests as typical PD motor function deficits. Even earlier, but often unrecognized, sensory and autonomic neurons succumb to αSyn and lipid overload, manifesting in sensory loss, chronic muscular, neuropathic and visceral pain and headache^40,41^. We have shown that sporadic PD patients have increased plasma concentrations of glucosylceramides, which was associated with a loss of thermal perception, mechanical hypersensitivity in Quantitative Sensory Tests, and high pain ratings^42^. The results suggested that accumulation of glucosylceramides may be common phenomenon in PD, not restricted to the about 5–15% PD patients carrying a pathogenic *GBA1* mutation^28^.

To obtain further insight into the pattern of glycosphingolipids and their impact on other lipids we used a back-translational approach and assessed lipidomic patterns in human PD plasma versus controls, patient derived primary fibroblasts, brain tissue of PD mice carrying a double PD-causative Pink1 deletion plus *SNCA* mutation (*SNCA* A53T) and in HT22 hippocampal neurons stimulated with preformed αSyn fibrils, and then we investigated effects of GlcCer in vitro on i) G-protein coupled receptors (GPCRs), ii) handling of preformed aSyn fibrils and iii) transcriptomic changes in vulnerable primary neurons.

GlcCer or HexCer were increased in all PD species/sites that were examined but GlcCer did not affect the uptake of αSyn fibrils or function of GPCRs, the latter suggested to be a target of glucosylsphingosines (GlcSph). Instead, exposure of primary sensory neurons to GlcCer 24:1 resulted in a transcriptomic switch reminiscent of cell responses to pathogen-derived glycolipids and suggests that extracellular GlcCer may mimic pathogenic threats. Since sensory neurons are a site of early premotor manifestations of PD and a source of αSyn spreading the observed mechanism may be relevant to the progression of the disease and potentially susceptible to therapeutic intervention.

## Results

### Increased plasma glucosylceramides in PD patients

**Fig. 1:**
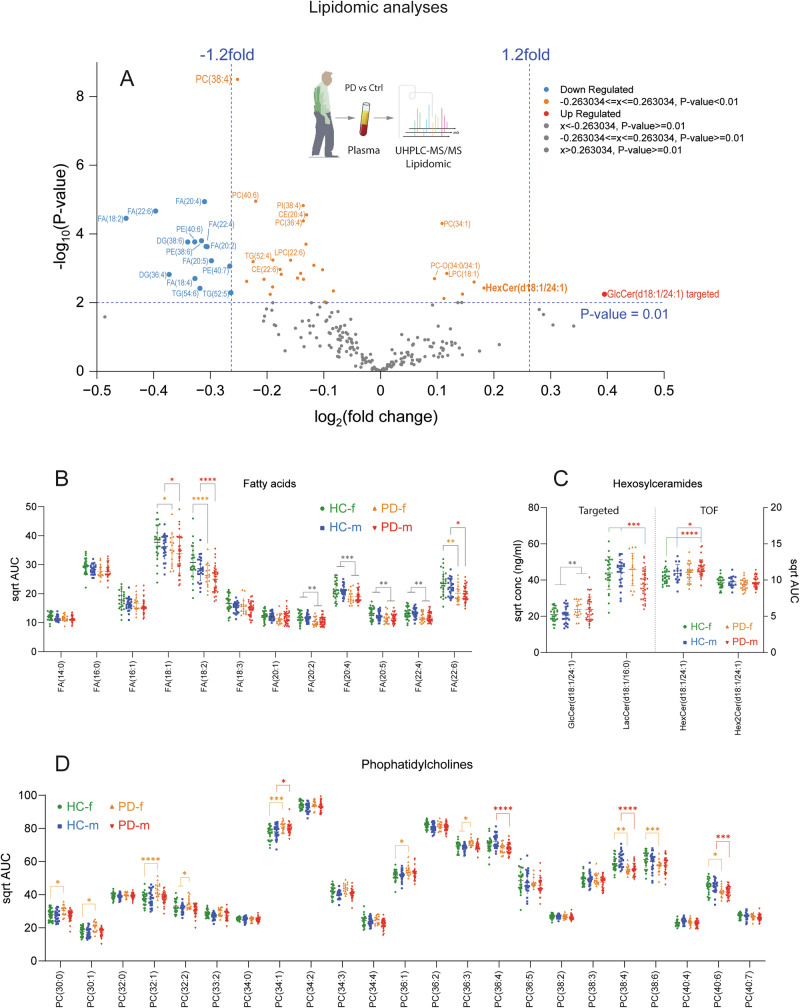
Plasma lipidomic analyses in PD patients versus healthy controls (HC). Patients (*n* = 16 f, 34 m) and controls (*n* = 25 f, 25 m) were age-matched <60-70+ years old at the time of blood sampling. Demographic details are shown in Supplementary Table 1. **A** Volcano plots show the Log2(fold change) on the x-axis versus the negative Log10 of the *t*-test *P*-value on the Y-axis. Increased lipids in PD are in red, reduced in blue. Lipids with statistically significant *P*-value but below the threshold for fold change are in orange. The Volcano plots reveals changes in FA, HexCer and PC which are detailed in (**B**–**D**). Please note that hexosylceramides (HexCer) represent GlcCer and GalCer. Hex2Cer mostly LacCer. **B** Fatty acids (FA) female and male PD patients and controls. **C** Hexosylceramides obtained by targeted and untargeted (TOF) lipidomic analyses. **D** Phospahtidylcholines (PC). The line is the mean, the whiskers show the SD. Each scatter is a subject. Data were submitted to 2-way ANOVA and subsequent posthoc analysis for each lipid species using a adjustment of alpha according to Sidak. *P < 0.05, **P < 0.01, ***P < 0.001, ****P < 0.0001. CAR carnitines, CER ceramides, CE cholesterol ester, DG diglycerides, FA fatty acids, HexCer hexosylceramides, LPC lysophosphatidylcholines, LPE lysophosphatidylethanolamines, LPG lysophosphatidylglycerols, LPI lysophosphatidylinositols, PC phosphatidylcholines, PE phosphatidylethanolamines, PD phosphatidylglycerols, PI phosphatidylinositols, SM sphingomyelins, ST sterols, TG triglycerides, UbiQ ubiquitin, –O ether bound.

**Fig. 2:**
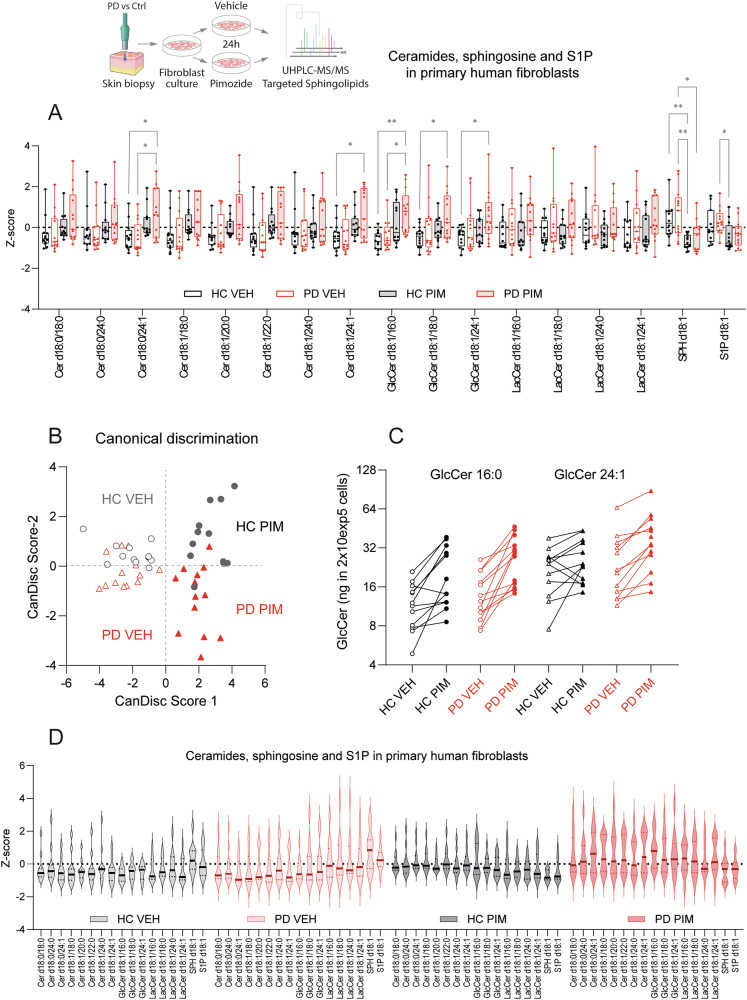
Ceramides and hexosylceramides in PD and HC primary fibroblasts upon stimulation with pimozide. Primary fibroblast cultures were obtained from 3 mm skin biopsies of the lower leg from *n* = 12 (9 f, 3 m) HC and *n* = 13 (4 f, 9 m) PD patients. Sphingolipids were analyzed by targeted LC-MS/MS from 2.5 ×105 primary human fibroblasts per sample, and concentrations in ng/ml were auto-scaled to have an common mean and variance of 1 (Z-score). Sub-confluent cultures were stimulated with 12.5 µM pimozide (PIM 12.5 µM = 1/5^th^ of EC50) or vehicle (1:10000 DMSO) and harvested at 24 h. **A** Box/scatter plots of sphingolipid z-scores. Pimozide treatment raised Cer 24:1 and GlcCer’s and reduced sphingosine (SPH d18:1) predominantly in PD-PHF. The box shows the interquartile range, the line is the median, whiskers show minimum to maximum, scatters are individual subjects. Statistics: 2-way ANOVA and subsequent *t*-tests with adjustment of alpha according to Sidak for the between subject factor (4 groups) *P < 0.05, **P < 0.01, ***P < 0.001. **B** Score plots of a canonical discrimination analyses using sphingolipid concentrations as input. Clusters of vehicle treated PHF are overlapping, but pimozide treated PD-PHF differ from HC-PHF. **C** Paired analysis of for the most abundant GlcCer 16:0 and GlcCer24:1. PD fibroblasts show a stronger PIM evoked increase. **D** Violin plots reveal stronger variance of sphingolipid levels in PHF of PD patients than controls. Data as in (**A**).

**Fig. 3:**
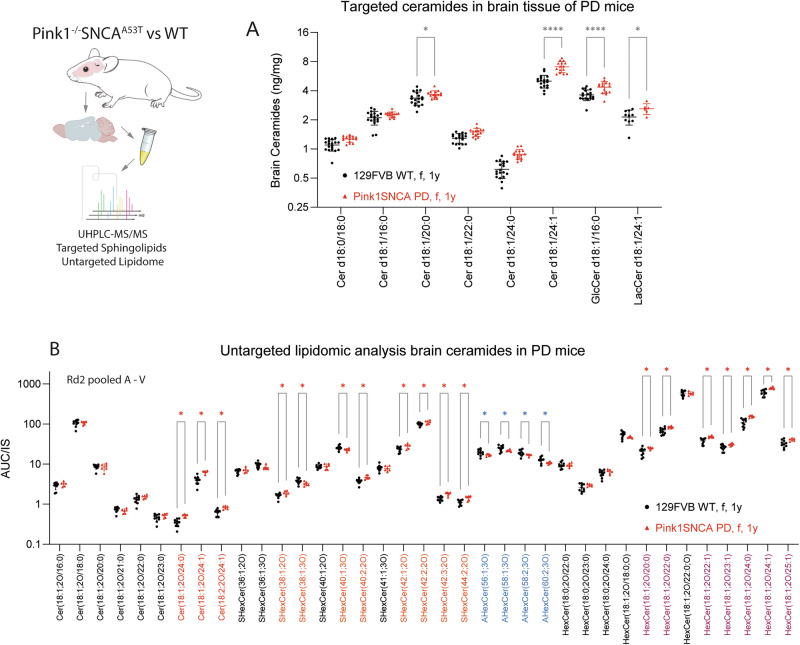
Ceramides and hexosylceramides in PD mouse brain. **A** Ceramides and hexosylceramides in brain tissue (cortex, subcortex, midbrain) in *Pink1*^*−/−*^*SNCA*^*A53T*^ and Sv129FVB wildtype control mice (age 50–60 weeks) analyzed by targeted LC-MS/MS analysis. Each scatter is one mouse. Data were submitted to 2-way ANOVA using the between subject factor “genotype” and the within subject factor “ceramide”, followed by posthoc *t*-tests with adjustment of alpha according to Sidak for genotype. *P < 0.05, ****P < 0.0001. **B** Ceramides and hexosylceramides in brain tissue (cortex, subcortex, midbrain) in *Pink1*^*−/−*^*SNCA*^*A53T*^ and Sv129FVB wildtype control mice (age 50–60 weeks) analyzed by untargeted UHPLC-MS/MS lipidomic screen. Data were analyzed using 2-way ANOVA with the between subject factor “genotype” and the within subject factor “ceramide”, followed by posthoc analysis for each lipid using the false discovery rate for adjustment of alpha. Heatmaps of top 50 regulated lipids are presented in Supplementary Fig S3.

**Fig. 4:**
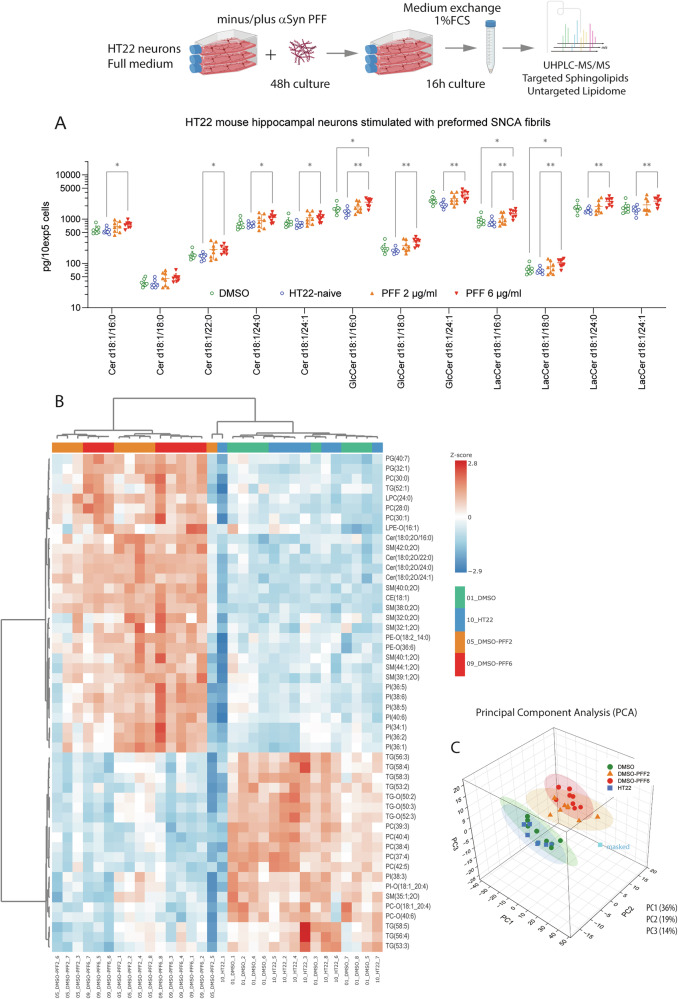
Preformed alpha synuclein fibrils (αSyn-PFF) increase ceramides in HT22 cells. **A** Ceramide and hexosylceramide concentrations in picograms per 100,000 HT22 mouse hippocampal neurons as assessed by targeted LC-MS/MS analysis. Cells were seeded in the absence or presence of PFF at 2 µg/ml or 6 µg/ml in culture flasks and grown for 48 h. Control cells were treated with the respective volume of DMSO or were left untreated (naïve HT22). Each group consisted in 8 replicates. Data were submitted to 2-way ANOVA and subsequent posthoc *t*-test using an adjustment of alpha according to Sidak for the between subject factor “treatment” (i.e. 4 groups). *P < 0.05, **P < 0.01. High PFF increased all ceramides. **B** Heatmap of top 50 regulated lipids in HT22 mouse hippocampal neurons as assessed by UHPLC-MS/MS lipidomic analysis. Cells were treated as explained in (**A**). AUC/IS values were square root transformed and auto-scaled to have a common mean and variance of 1 (z-scores). Lipids were clustered according to Euclidean distance metrics using the Ward method. **C** Principal Component Analysis of HT22 lipidomic data showing a 3D-score scatter plot of the first three PC. Each scatter shows one sample. The circles are 90% confidence elipsoids.

**Fig. 5:**
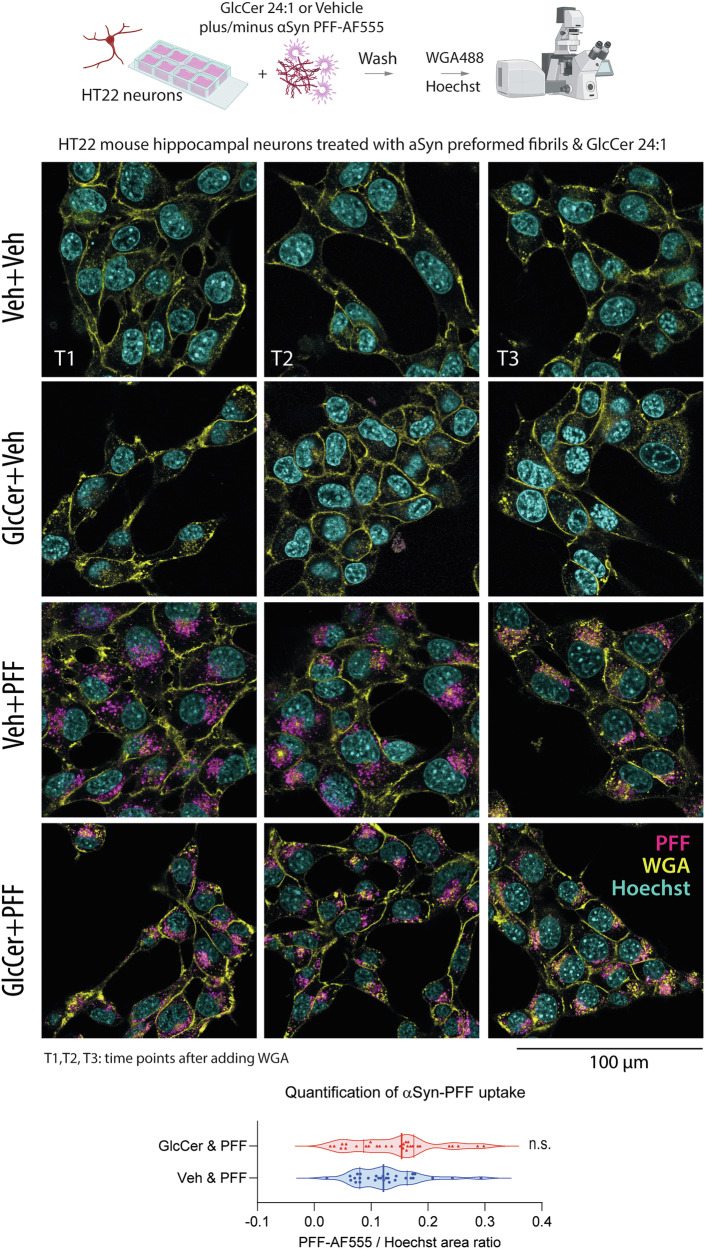
Cellular uptake of preformed alpha synuclein fibrils (αSyn-PFF) in HT22 cells. Immunofluorescent studies show the uptake of AF555-labeled αSyn preformed fibrils in HT22 mouse hippocampal neurons. Pretreatment with GlcCer24:1 (1 µM) had no significant impact on the PFF-AF555 fluorescence uptake in HT22 cells (pink). Wheat germ agglutin WGA-AF488 (yellow) is used as counterstain of plasma membranes (early time point) and endolysosomes (late time point). Hoechst-33342 is used as nuclear stain. Additional zoom-in images are shown in Supplementary Fig. S5.

In previous studies, we^42^ and others^43,44^ have observed increased plasma concentrations of ceramides and glucosylceramides or glucosylsphingosines in patients with idiopathic PD who were not carrying *GBA1* mutations that are associated with mal-folding or dysfunction of the *GBA1* gene product, lysosomal glucocerebrosidase alpha (GCase). To gain further insight into PD-associated ceramide pathology we explored and compared lipidomic patterns in plasma (Fig. 1) and primary fibroblasts of PD patients (Fig. 2), in brain tissue of PD-mice (Fig. 3), and in HT22 mouse hippocampal neurons loaded with αSyn preformed fibrils (Figs. 4 and 5).

Plasma lipidomic studies of 50 PD patients versus 50 age-matched healthy controls (HC) revealed increased hexosylceramides in PD, particularly GlcCer24:1 (Fig. 1A, Supplementary Fig. S1), stronger in male than female PD patients (Fig. 1C), whereas a number of mono- and poly-unsaturated fatty acids were reduced without differences between sexes (Fig. 1B). In addition, phosphatidylcholine species (PC) shifted from long chain poly-unsaturated PC species to shorter mono-unsaturated PCs (Fig. 1D) without overall change of summed PCs, which agrees with a recent study where a set of serum lipids including PC species allowed for a better prediction of PD progression/outcome than age, sex and risk gene LRRK2 mutation status^45^. PCs are connected with GlcCer via the phospholipid translocase *ATP10* (*ATP10A, 10B and 10D*), which flips PC in exchange with GlcCer in the plasma membrane, and contributes thereby to membrane dynamics^23,46,47^, and is considered as PD risk gene^23^. A recent study shows that ATP10 rather than GCase accounts for increased GlcCer in PD^48^.

Plasma lipidomic data and metadata are available at https://www.ebi.ac.uk/biostudies with the accession number S-BSST1880. https://www.ebi.ac.uk/biostudies/studies/S-BSST1880?key=a0dc6f53-0365-410a-96e3-d83e51a04282.

### Increased glucosylceramides in PD primary fibroblasts upon stimulation with pimozide

GlcCer are generated in the ER, from there transported and inserted into the plasma membrane and membranes of organelles, and they are degraded in the lysosomes^49^. Considering the autophagolysosomal pathophysiology of PD the most likely mechanism underlying increased GlcCer in plasma is mal-degradation in the lysosomes^12,14,50,51^. To study this aspect we used primary patient derived fibroblasts that were stimulated with pimozide^52^.

Patient derived primary fibroblasts are considered a viable model for studying neurodegenerative changes due to their metabolic and biochemical relationships with neurons^53^. In particular, the phenotype of skin fibroblasts of late-onset sporadic PD subjects recapitulated features of iPSC derived midbrain dopamine neurons from the same patient, including PD-associated changes in morphology, mitochondrial function, and autophagy^54^. Fibroblasts of PD patients express about 5-10 fold more αSyn^55^ and are used for early diagnosis of prodromal symptoms^56^. Further, sphingolipid changes found in PD fibroblasts precipitated the aggregation of αSyn^57^. Primary patient derived fibroblasts were therefore chosen to further study GlcCer alterations.

To mimic PD-like lysosomal dysfunctionality, primary human fibroblasts (PHF) of 13 PD patients and 12 HC were stimulated with pimozide, which is a lysosome-tropic, antipsychotic drug. As a weak base (pKa 8.36) pimozide is trapped in lysosomes but does not cause major lysosomal alkalinization and therefore does not cause complete disruption of lysosomal functions like chloroquine^52^. At high concentrations, its accumulation in lysosomes can cause lysosomal membrane permeabilization and lysophagy^15^ but at lower concentrations it mainly interferes with lysosomal (sphingo)-lipid metabolism that manifests in an increase of ceramides and GlcCer in cell lysates^15,52^. It was therefore an ideal stimulus to assess subtle differences of lysosomal functions in PD versus control fibroblasts.

The IC50 in primary human fibroblasts (PHF) of pimozide-evoked cell death was 90-100 µM (Supplementary Fig. S2). To assess effects on lipid homeostasis, PHF were stimulated for 24 h with 1/8^th^ of the IC50 (i.e. 12.5 µM). At baseline, there was no difference between PD and HC fibroblasts in any of the analyzed sphingolipids (Fig. 2). Pimozide increased ceramides and reduced sphingoid base sphingolipids (sphingosine, S1P) in both PD and HC fibroblasts in line with previous observations in tumor cells^15^, but the pimozide-evoked increase of glucosylceramides was stronger in PD fibroblasts (Fig. 2A, C). Canonical discrimination analyses using all sphingolipids as input was able to distinguish PD-PIM versus HC-PIM fibroblasts (Fig. 2B). Further, violin plots of Z-transformed sphingolipids revealed higher variability of PD fibroblasts as compared with HC fibroblasts (Fig. 2D). The results suggest higher vulnerability of PD-fibroblasts towards a pimozide-evoked raise of lysosomal pH possibly because PD fibroblasts have a higher lysosomal burden of αSyn^55^ and therefore lower capacity to adjust lysosomal hydrolase activity.

Fibroblast sphingolipid raw data and metadata are available at https://www.ebi.ac.uk/biostudies with the accession number S-BSST1881. https://www.ebi.ac.uk/biostudies/studies/S-BSST1881?key=4e8ae53f-17f0-4179-80ff-40392e418270.

### Increased ceramides and hexosylceramides in PD mouse brain

Plasma hexosylceramides are maintained and replenished from peripheral and central sources. The relative contribution is unknown. Elevated plasma levels in PD patients suggested disease-associated metabolic changes, but plasma levels represent the extracellular compartment and may not necessarily directly reflect brain tissue. Therefore, we used PD-mice to get direct insight into brain ceramides. As explained in the description of the PD mouse model, we opted for *Pink1*^*−/−*^*SNCA*^A53T^ mice because they combine features of human PD including a premotor disease with sensory symptoms and development of spontaneous motor deficits.

Targeted (Fig. 3A) and untargeted (Fig. 3B) lipidomic studies revealed increased ceramides in the brain of double mutant *Pink1*^*−/−*^*SNCA*^A53T^ mice compared with wildtype Sv129-FVB control mice, in agreement with previous ceramide studies in Pink1^−/−^ mouse brain^58^. Importantly, *Pink1*^*−/−*^*SNCA*^A53T^ mice had no clinical symptoms of PD motor disease at the time of tissue sampling at ~12 months of age. Ceramides, hexosylceramides (GlcCer or GalCer) and sulfatides (SHexCer) were increased in *Pink1*^*−/−*^*SNCA*^A53T^ brains whereas acetyl-HexCer (AHexCer) were decreased (Fig. 3B). Lipidomic analyses further revealed increased diacylglycerols (DG) and lysophosphatidylethanolamines (LPE) in *Pink1*^*−/−*^*SNCA*^A53T^ brains (Supplementary Fig. 3). DGs are normally low in the brain, and high brain DGs have been suggested to indicate CNS disease^59^. LPEs are contained in Lewy bodies and likely precipitate αSyn aggregation, depending on C-chain length and saturation^43^. Lipidomic heat maps of candidate lipids also show a switch of PC species not equal but corresponding to the patterns in human plasma, mainly a decrease of long-chain PC-O and increase of shorter PCs, mostly with low saturation.

Brain sphingolipid and lipidomic and metabolomic raw data and metadata are available at available at https://www.ebi.ac.uk/biostudies with the accession number S-BSST1888. https://www.ebi.ac.uk/biostudies/studies/S-BSST1888?key=6230dc37-d998-4eca-bf0f-3502f7164655.

### Increased ceramides in HT22 neurons upon ingestion of preformed αSyn fibrils

Mutant *GBA1* and αSyn mutually increase neuronal damage likely converging on lysosomal collapse^22,50^ and mitochondrial toxicity^36^. To mimic the αSyn part, HT22 mouse hippocampal neurons were exposed to active preformed αSyn fibrils, which were supplied via the culture medium. Using confocal live imaging microscopy it was confirmed that HT22 cells ingested PFF, thus creating PD-like HT22. HT22 cell were chosen as a model because of their well-established sensitivity to PD-inducing pro-oxidative agents, expression of dopamine receptors, clathrin endocytosis machinery and the observed reliable uptake of αSyn-PFF.

Lipidomic analyses of such PD-like HT22 showed that the exposure and ingestion of αSyn fibrils increased ceramides and hexosylceramides. The higher dose of 6 μg/ml caused stronger changes than the low dose of 2 µg/ml (Fig. 4A). αSyn fibrils alone had no effect on cell viability as assessed by WST-1 assays (Supplementary Fig. 4A, B), nor did it consistently affect polar metabolites. At high concentration, αSyn-PFF treatment resulted in an increase of LDH activity in supernatants in some cultures (Supplementary Fig. S4C), suggesting damage of the cell membrane (cytosolic LDH) or lysosomal exocytosis (lysosomal LDH). Lipidomic analyses (Fig. 4B, C) revealed that ceramides were among the top upregulated lipids exposed to αSyn PFF. In agreement with mouse brain and human plasma, PFF ingestion was associated with an increase of short-chain PC but decrease of long-chain PC (Supplementary Fig. S4C). In addition, heatmap analyses showed a strong increase of phosphatidylinositol (PI) species which had not been observed in mouse brain or human plasma. Score plots of a principal component analysis showed a distinction of PFF-treated versus not-treated cells, but no PFF dose-dependent difference.

HT22 lipidomic and metabolomic data and metadata are available at https://www.ebi.ac.uk/biostudies with the accession number S-BSST1897. https://www.ebi.ac.uk/biostudies/studies/S-BSST1897?key=d2a20a65-a88f-44a4-9654-672a72f8a9ac.

### No significant increase of PFF ingestion in the presence of GlcCer24:1

It has been suggested that *GBA1* deficiency exaggerates the exosomal spreading of αSyn^60,61^ likely because the release of exosomes which are enriched in GlcCer^62^ is a compensatory mechanisms to get rid of excess GlcCer. Spreading also requires subsequent uptake in recipient cells. Therefore we studied in a series of confocal live imaging experiments if extracellular GlcCer 24:1 enhanced the uptake or distribution of αSyn PFF in HT22 hippocampal neurons. The plasma membrane was visualized with fluorescent wheat germ agglutinin (WGA-AF488). During the observation time, WGA was taken up and moved to the lysosomes (Supplementary Fig. S5 WGA co-staining with LysoID®). Hence, in the first images, WGA is localized mostly at the outer membrane (left panel in Fig. 5), whereas it co-segregates with αSyn PFF in the lysosomes at late images (right panel, Supplementary Fig. S5 co-staining with LysoID®). The middle panel is a time point in between. GlcCer 24:1 treatment had no overt effect on the quantity of ingested αSyn PFF but the cell size was more variable in GlcCer 24:1 plus PFF-treated cell cultures and some cells had thin cell-to-cell contacts (Fig. 5 bottom row left image, Supplementary Fig. S5 Zoom-in) and WST-1 assays showed that the combination of GlcCer 24:1 plus αSyn PFF for 48 h increased the percentage of non-viable cells (Supplementary Fig. S4).

### No activation of G-protein coupled receptors by GlcCer 24:1 and 18:1

**Fig. 6:**
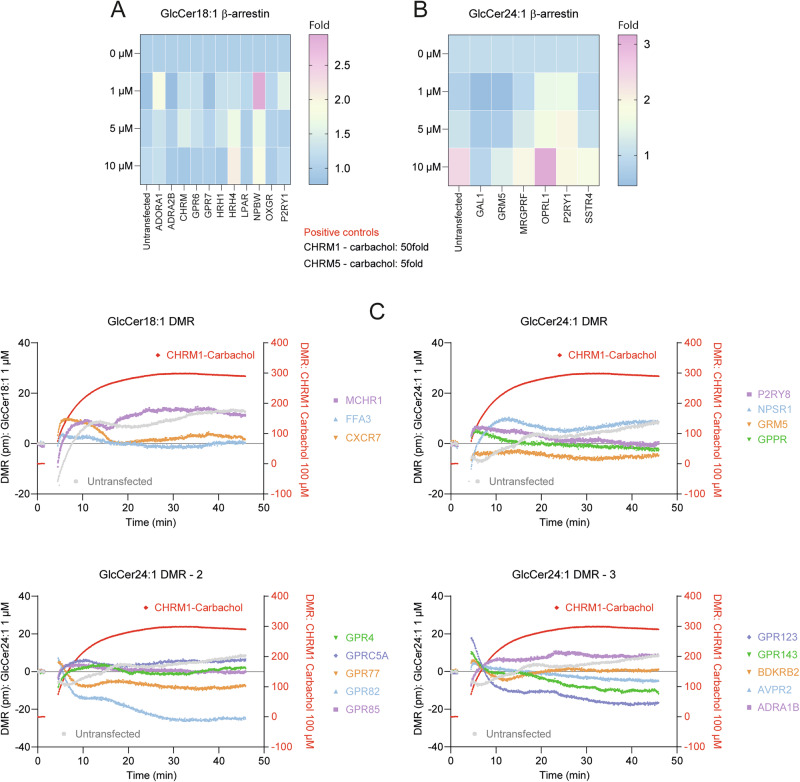
GlcCer18:1 and GlcCer24:1 do not activate heterologous expressed G-protein coupled receptors. **A** Beta arrestin-based screening of GPCR activity in a heterologous expression model in COS cells upon stimulation with GlcCer 18:1 versus vehicle at 1, 5 and 10 µM. The heatmap shows the mean of four replicates for candidate GPCR selected from an initial screen (330 GPCRs, 8 neg and 4 × 2 pos controls). Details in Supplementary Fig. 1. **B** As in A but stimulation with GlcCer 24:1 C: Dynamic Mass Redistribution (DMR) analysis of candidate GPCRs stimulated with GlcCer 18:1 or GlcCer 24:1 at 1 µM. DMR measures a ligand-induced shift in resonant wavelength in picometers. Three candidate GPCR were tested with GlcCer 18:1 (upper left) and 14 with GlcCer 24:1. Carbachol activation of muscarinic receptor CHRM1 was used as positive control (right Y-axis). MCHR1 melanin-concentrating hormone receptor 1, FFA3 free fatty acid receptor 3 (GPR41), CXCR7 CXC-type chemokine receptor 7, GPR4 G-protein coupled receptor 4, pH-sensing, GPRC5A orphan GPCR, alias: retinoic acid-induced protein 3 (RAI3), GPR77 C5a anaphylatoxin chemotactic receptor, GPR82 and GPR85 both orphan, P2RY8 P2Y purinoceptor, NPSR1 neuropeptide S receptor 1, GRM5 metabotropic glutamate receptor, GPPR gastrin releasing peptide receptor, GPR123 adhesion GPCR, GPR143 ocular albinism type 1 (OA1), receptor for tyrosine, L-dopa and dopamine, BDKRB2 bradykinin receptor B2, AVPR2 arginine vasopressin receptor 2, ADRA1B alpha-1B adrenoreceptor.

High levels of GlcCer in extracellular fluids (plasma) suggest that GlcCer get into contact with the outer membrane, membrane receptors, transporters or channels. We have previously observed that GlcCer in culture medium increased stimulated calcium fluxes in a fraction of neurons, but not per se without additional stimulus^63^. GlcCer are important components of membrane microdomains and hence involved in the regulation of membrane receptor insertion and function^64,65^. Considering the importance of dopamine receptors in PD and previous reports showing activation of serotonin receptors with GlcSph we assessed putative direct effects of the top PD-associated GlcCer candidates in human plasma, GlcCer18:1 and GlcCer24:1, on G-protein coupled receptors via beta-arrestin screening and dynamic mass redistribution (DMR) assays. Compared with the positive control (carbachol, muscarinic GPCR), GlcCer18:1 and GlcCer24:1 had no effect on any of the screened GPCRs (Fig. 6A, B; Supplementary Fig. S6 showing replicates) at a reasonable concentration range up to 10 µM. At 10 µM, GlcCer24:1 increased beta-arrestin binding up to 3-fold in HTLA cells with heterologous expression of OPRL1 (opioid related receptor, nociceptin/orphanin FQ). The result was reproducible in quadruple replicates (Supplementary Fig. S6) but still weak compared with the positive controls.

### GlcCer 24:1 causes inflammatory transcriptional response in primary mouse neurons

**Fig. 7:**
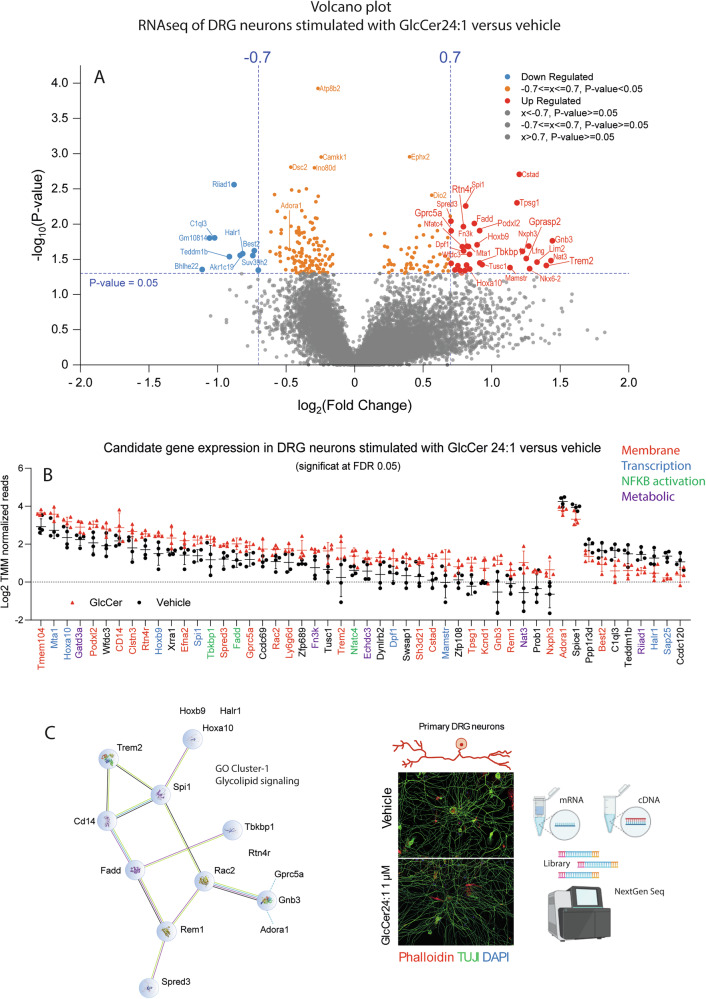
GlcCer24:1 of primary neurons triggers upregulation of genes involved in GO “response to glycolipid”. **A** Volcano plots of regulation of mRNA expression assessed by RNAseq in primary sensory neurons of the dorsal root ganglia from adult mice treated with 1 µM GlcCer24:1 versus vehicle. The x-axis shows the Log2(Fold change) of TMM-normalized counts. The y-axis shows the negative logarithm of the *t*-test *P*-value. The data are from *n* = 4 cultures per condition. Upregulated genes are shown in red, downregulated in blue. **B** Candidate genes were selected according to the FDR-adjusted *P*-value and are sorted according to abundance and presented as TMM-normalized reads (trimmed mean of m values). **C** Candidate genes were submitted to GO and pathway enrichment analysis using DAVID, STRING and Panther consistently showing an enrichment of genes involved in “response to glycolipid”.

The loss of motor functions in PD is often preceded with non-motor symptoms originating from manifestations of the disease in the peripheral somatosensory and autonomous nervous systems^66,67^. The premotor phase may last for years and causes (among others) PD-associated sensory neuropathy^68^ with sensory loss and pain^69–71^, and restless leg syndrome^72^. In “body-first” PD, αSyn spreading originates from the peripheral nervous system^73–75^. Somatosensory neurons are therefore in many cases the site of the disease onset where the progression may still be responsive to disease-modifying intervention. We have shown previously that sensory loss and pain is associated with high GlcCer in plasma (PD patients) or DRGs and sciatic nerve tissue (mice) and that treatment of dorsal root ganglia (DRG) neurons with GlcCer 24:1 leads to an exaggerated calcium influx upon stimulation^42,63^. Therefore, we used adult DRG neurons to gain further insight into the effects of GlcCer using mRNA sequencing to reveal transcriptomic changes that occur when DRG neurons are exposed to GlcCer 24:1 (Fig. 7).

RNAseq revealed an upregulation of a number of membrane-associated genes (GO CC: membrane) and overrepresentation of genes involved in the “response to glycolipids” (GO BP or similar terms such as “response to LPS”, “response to pathogens”). The results suggest that extracellular GlcCer can induce inflammatory responses similar to those induced by pathogens. The results agree with a recent study showing that glucosylceramide accumulation due to GCase deficiency caused macrophage activation and neuroinflammation^76^. Candidate genes, gene descriptions and GO studies are presented in a Supplementary Excel file. RNAseq data have been deposited as GEO dataset with the provisional accession number GSE262573.

To review GEO accession GSE262573.

Enter token clytuucuxjupzmj into the box.

## Discussion

Lewy bodies are primarily composed of α-synuclein fibrils intertwined with lipids. Mutations in genes responsible for lipid degradation or transport, such as *GBA1, ATP13a2 and ATP10b*, have been identified as factors that increase the risk of PD^23,77^. Although mechanistically complex, lipidomic research suggests that an accumulation of glycosylated sphingolipids may enhance the pathogenicity of αSyn^3,4,78^ by precipitating the formation of αSyn fibrils^6^. In the present study, we show that glucosylceramides are increased in the plasma of PD patients, in the brain of PD mice, in primary human fibroblasts of PD patients treated with pimozide and in HT22 hippocampal neurons after ingestion of preformed αSyn fibrils, together strongly suggesting that αSyn interferes with metabolic homeostasis and/or release of hexosylceramides. The predominant candidate was GlcCer 24:1. Except AHexCer in the mouse brain, all GlcCer (HexCer) species were increased in human PD, or PD-models. However, GlcCer 24:1 (and GlcCer 18:1 in some experiments) had little effect on cell viability, morphology, ingestion of αSyn, or GPCR activation which agrees with a recent study that shows high plasma GlcCer in association with PD but not in a causative manner. The authors concluded that GlcCer per se was not pathogenic^48^ but changes of GlcCer need to be considered in the context of more complex changes of lipids that are connected via metabolic and transport pathways. In particular, we and several previous studies observed changes in a number of phosphatidylcholine (PC) species^43,79–82^ whose specific functions are presently mostly elusive, but determine the secondary structure of αSyn oligomers^83,84^ and are intricately linked to endolysosomal transfer and membrane insertion of GlcCer via flippases^77,79,85^. Nonetheless, alterations of HexCer are the most robust finding in PD and models of PD and our RNAseq studies have shown that GlcCer 24:1 treatment of primary neurons elicited a pro-inflammatory response. In support, a recent study in a PD fly model showed that GlcCer caused cellular immune activation^76^.

Considering the close association of glucosylceramides with the risk and the course of PD we asked how elevated glucosylceramides might contribute to an unfavorable course of PD. We considered three putative mechanisms: (i) by activation or inhibition of GPCR signaling (based on studies with hexosylsphingosines^86,87^), (ii) by facilitating αSyn uptake and (iii) by causing a glycolipid-like immune response. We found no evidence for the first two hypotheses but some support for the third.

In β-arrestin-based GPCR screening assays, GlcCer 24:1 induced a dose-dependent increase of β-arrestin binding in *OPRL1* positive clones (nociception/orphanin-FQ) up to 3-fold. However, the effect was weak compared with the positive control carbachol, which increased the β-arrestin signal up to 50-fold upon binding to muscarinic choline receptors (*CHRM1*). In DMR-based GPCR screening, GlcCer had no effect. We infer that GlcCer 24:1 (and 18:1) do not activate or inhibit GPCRs at concentrations which might be reached in extracellular fluids. Importantly, GlcCer 24:1 or 18:1 had no direct effect on dopaminergic receptors which could have been a putative disease-relevant site of action. It is of note that glucosylsphingosines (GlcSph), which are the deacylated (lyso-) form of glucosylceramides, were found to activate serotonin receptors^88^ and that glucosylsphingolipids may indirectly affect GPCR by changes of membrane composition and lipid rafts^89^.

Both, GlcCer and GlcSph accumulate in brain and peripheral organs in Gaucher disease^90^, but GlcSph are preferred as “biomarkers” in Gaucher disease^91^ because *GBA1* deficient macrophages (Gaucher cells) were reported to accumulate predominantly the lyso-glycosphingolipid^92^, which however does not confer more or less toxicity to the acylated or deacylated version for neurons in the context of neuronal type Gaucher disease and PD, but might explain why GlcSph has gained more attention^93–95^ and is analyzed for newborn Gaucher screening^96,97^. But GlcCer and gangliosides rather than GlcSph were found to precipitate αSyn oligomerization in acidic environments in vitro^98^.

αSyn monomers normally bind to the surface of synaptic vesicles^99,100^. Their oligomerization properties enable vesicle tethering but αSyn is not essential for synaptic vesicle cycling and release^101^. However, excess or mutant or phosphorylated αSyn adopts beta sheet rich conformations which are prone to oligomerization, further aggregation and fibril formation and spread like prion-like proteins^9,102,103^. αSyn uses several means of cell-to-cell spreading^104,105^ including transsynaptic transfer from neuron to neuron, traveling through tunneling nanotubes^8,51,106^, packaging into and release of extracellular vesicles which can reach far distant target cells and are taken up endocytosis^7,107^. Further, αSyn can be expelled via lysosomal exocytosis likely together with GlcCer thereby creating a hostile microenvironment^108^. GlcCer are also components of exosomal membranes^109^ and may assist in packaging of αSyn into exosomes^110^, and define the target cells^111^. Our experiment created a neuronal microenvironment with excess free non-exosome-packed αSyn fibrils (plus/minus GlcCer24:1) and high extracellular GlcCer, a situation that occurs when αSyn fibrils or aggregates are expelled from cells via exocytosis or lysosomal exocytosis. Because of the excess of PFF, all neurons ingested the fibrils, and our experiment did not allow for live-observation of αSyn-PFF spreading from one population of neurons to another. In extracellular fluids, GlcCer likely bind to albumin or other proteins, but the relative contribution of exosomal, albumin/protein-bound and free GlcCer is unknown. Under the experimental conditions of this study, GlcCer did neither increase the uptake of αSyn-PFF nor the accumulation in lysosomes. However, results may differ in mixed cultures that include immune cells and glia, and/or in compartmentalized cultures with localized preformed fibril (PFF) seeds.

In myeloid immune cells, GlcCer have been described as ligands of Mincle (*Clec4e*)^112,113^, which is an inducible C-type lectin and innate immune receptor of macrophages, neutrophils and dendritic cells that senses glycolipids of the mycobacterial cell wall (trehalose 6,6’-dimycolate, known as cord factor)^114,115^ and other glycolipid species derived from pathogens or damaged and dead cells^116–118^. Mincle (*Clec4e*) was weakly expressed in our primary sensory neuron cultures (RNAseq studies). For comparison, Clec4e total read counts ranged from 11 to 70, whereas total read counts of marker genes for DRG neurons were in the range of 100–4000, e.g. *TRPA1* ~100, *TRPV1* ~300, *Scn10a* (Nav1.8) ~∼800, *TAC1* (Substance P) ~4000, and relevant lipid-sensitive GPCRs were e.g. *LPAR5* ~∼15, *PTGER4* (EP4) ~∼50, *CNR1* (CB1) ~∼300, *S1PR1* ~∼70 and *S1PR3* ~∼600. Hence, *Clec4e* expression was low but still in a range where its activation may result in measurable effects. In line with this reasoning we found that treatment of primary sensory neurons with GlcCer 24:1 resulted in a transcriptional response similar to that observed by stimulation of cells with bacterial glycolipids. Gene ontology analyses of upregulated genes showed an enrichment of terms like “toll-like receptor signaling”, “positive regulation of innate immune response”, “pattern recognition receptor signaling”. The data suggest that high extracellular GlcCer may induce an innate immune response, which is adequate for the defence against mycobacterium but may contribute to neuroinflammation if it were to occur in the brain. The latter is supported by a study where GlcCer stimulated microglia phagocytosed living neurons^119^. We used primary sensory neurons because they are a site of early PD manifestations and sensitive to GlcCer but it needs to be assessed in future studies if GlcCer elicits similar innate immune responses in neurons of the CNS.

C-type lectin activation leads to spleen tyrosine kinase (Syk) mediated downstream signaling involving phospholipase C, protein kinase C, MAP kinases and nuclear factor kappa B (NFκB). Considering that the most frequent loss-of-function mutation, *GBA1* N370S, that causes Gaucher disease confers resistance to tuberculosis in a zebrafish model^120^ it is tempting to speculate that GlcCer-mediated Mincle/Clec4e activation is protective against pathogens but possibly harmful if it sustains a microglial attack of neurons. It has been shown that Syk inhibitors reduce neuroinflammation, but so far only in models of stroke or vascular brain injury^121^, not yet for Gaucher disease or *GBA1*-associated Parkinson’s disease. It is of note that a recent study suggested to add a Mincle activating adjuvant to αSyn-peptides to enhance the efficacy of active PD vaccination^122^. The vaccine consisted in a glucan-αSyn conjugate, i.e. not a lipid-conjugate but the strategy reduced fibril propagation in an in vivo synuclein-seeding-spreading model. Hence, although the molecular target of GlcCer remains unidentified its immune-stimulating effect might be beneficial if it could be harnessed to enhance fibril elimination.

The present study has limitations. It focused on GlcCer in non-GBA1-associated PD, but it would have been valuable to include some confirmed GBA1 mutant patients and GBA1-mutant mice for comparison as “positive” control, particularly because we did not find quantifiable glucosylsphingosine species in our lipidomic analysis, which are supposed to be biomarkers for Gaucher disease. Although the frequency of PD-associated GBA1 mutations is low in Germany, it is a limitation that our patients were not genotyped for GBA1 or other PD-associated genes that affect lipid metabolism or transport. Hippocampal mouse HT22 neurons and primary mouse DRG neurons are valuable models for PD research but human CNS neurons may respond differently to PFF or GlcCer which needs to be assessed in human neurons or organoids in future studies.

In summary, we show that extracellular GlcCer is increased in PD and in PD models likely originating from defective lysosomal functions and the data suggest that extracellular GlcCer may induce an innate immune response in peripheral sensory neurons. Such immune activation may sustain a proinflammatory state but may also attract glia and immune cells to remove extracellular αSyn.

## Methods

### PD patients versus age-matched healthy controls

PD patients, diagnosed using ICD10 diagnostic criteria for Parkinson’s Disease, were consecutively recruited from the Movement Disorders Department of Neurology of the University Hospital in Frankfurt am Main, Germany as described^42^. Aged healthy control subjects were consecutively recruited from spouse and friends of PD patients and from the outpatient stroke unit of the University Hospital in Frankfurt am Main. Demographic data of patients and controls have been shown in detail in ref. ^42^. PD disease severity was assessed according to the Hoehn Yahr rating scale in “ON” periods for patients who experienced substantial disease fluctuations (16 out of 50 patients). Patients were not routinely genotyped for PD-associated *GBA1* mutations because the frequency in Germany is low (5-10%). Informed written consent was obtained from all subjects. The study was approved by the Institutional Ethics Committee of the University Hospital of Frankfurt (Approval #458/16, date 13.01.2017, amendment 01.10.2018). Data acquisition and blood sample collection adhered to the Declaration of Helsinki. Demographic data are presented in Supplementary Table 1.

### Skin biopsy and primary fibroblast culture of PD patients and controls

After local anesthesia by intradermal injection of lidocaine a 3 mm skin punch biopsy was obtained from the skin of the lower leg 10–15 cm above the ankle. The biopsy was placed in ice-cold DMEM medium for transport. Culture dishes (6-well plates) were prepared by coating the surfaces with FBS and dried under the clean bench. The biopsy was placed in a culture dish within 1 h and cautiously covered with fibroblast medium (low glucose DMEM (100 mg/l), 10% FBS, 1x MEM Non-Essential Amino Acids, 2 mM glutamine, 1% PenStrep) and fibroblasts were allowed to grow for 3–4 days. Fibroblasts were harvested with trypsin and sub-cultured in T75 flasks in fibroblast medium or in DMEM high glucose (5 g/l), 10% FBS, 1% PenStrep. Targeted lipidomic analyses were obtained from naïve cultures and upon stimulation with pimozide versus vehicle. WST-1 assays and immunofluorescence analyses were used to assess EC50 values of pimozide in PHF.

For lipidomic analyses, primary human fibroblast (PHF) cultures were stimulated with 1/8^th^ of the EC50 of pimozide (i.e. 12.5 µM; Merck, #573110-100MG) or vehicle (DMSO 1:10000) for 24 h, in two rounds, each consisting in 25 PHF cultures (13 PD, 12 HC cultures) of matched HC and PD patients. Sub-confluent cultures were split and plated on 10 cm culture dishes, each two for DMSO (Control) and Pimozide. Cells were allowed to attach and grow for 24 h, before treatments were added and cells grown for another 24 h. Cells were washed 3 × in 1× PBS, harvested by trypsinization, pelleted, washed, and adjusted to 250,000 cells per cell pellet which was frozen at −80° until lipid analyses.

Demographic data of PFB donors are presented in Supplementary Table 2.

### Pimozide IC50 in primary human fibroblasts

WST-1 and sulforhodamine B (SRB) cell viability/cell death assays were used to define stimulating but non-toxic pimozide concentrations and conditions. PHF were seeded in 100 µl medium/well of a 96 well plate, 5000 per well and allowed to grow for 24 h. The medium was replaced with full medium containing Pimozide at concentrations ranging from 1 to 256 µM (log2 scale 1–8 µM) or 1:10000 DMSO (=vehicle). The next day, 10 µl WST-1 reagent (Merck #11644807001) was added to each well and cells were incubated for 60 min at 37 °C. Formation of the formazan dye, which relies on the metabolic activity was then measured via its absorbance in a multi-well spectrophotometer (TECAN Infinite F200 Pro, RRID: SCR_020543) at 405 nm and 620 nm (reference). Subsequently, the reaction was stopped by adding 20 µl 50% trichloroacetic acid, TCA (w/v) to a final concentration of 10% TCA for 1 h at 4 °C. Wells were washed with distilled water 7-times and were dried. To quantify cell mass, fixed cells were stained by adding 100 µl 0.4% (w/v) sulforhodamine B (SRB) in 1% acetic acid for 30 min at room temperature under shaking. Wells were washed with 1% acetic acid 5-times and cells were lysed by adding 250 µl 10 mM Tris/HCl pH 10.5 for 10 min at room temperature. Absorbance of SRB at 540 nm was measured with TECAN plate reader. The percentage of viable cells compared with DMSO was plotted versus the log2 concentration and fitted with a inhibitory sigmoidal Emax model in GraphPad Prism 9 or 10 (RRID:SCR_002798).

The survival of HT22 cells in the presence of GlcCer24:1 or vehicle and AF555-labeled αSyn-PFF was tested using the WST-1 reagent (Merck #11644807001) as described above. HT22 cells were seeded in 96 well plates (4000 cells/well) and treated with 1 µM GlcCer24:1, with or without αSyn-PFF. After 48 h of incubation, 10 µl of the WST-1 reagent was added to the cells and after a 60 min incubation at 37 °C the absorbance was measured at 450 nm and 620 nm (reference) in a multi-well spectrophotometer as above. Subsequently, the SRB assay was performed.

### Double mutant *Pink1*^*−/−*^*SNCA*^A53T^ Parkinson-model mice

Homozygous *Pink1*^−/−^ plus *SNCA*^A53T^ double mutant mice and Pink1^−/−^ single mutant mice were generated as described in refs. ^63,123^ by crossing *Pink1*^−/−^ mice (MGI:3850370) with PrPmtA mice expressing human mutant *SNCA* A53T (mtA) under the prion promoter (PrP) (MGI:3723258) and then, interbreeding the littermates. Mice are available from Jackson lab as cryopreserved sperm (FVB;129-Pink1^tm1Aub^ X Tg(Prnp-SNCA*A53T)^AAub/J^; Strain #:017678). Double mutant mice are referred to as *Pink1*^*−/−*^*SNCA*^*A53T*^ or in short, *Pink1SNCA*.

We used *Pink1*^*−/−*^*SNCA*^A53T^ double mutant mice because unlike other PD models, they develop spontaneous PD-like motor symptoms with an onset at >15 months of age and a frequency of about 20-30 percent^123^ and they combine the complex PD pathophysiology of pro-oxidative and dysfunctional autophagolysosomal pathways^12,124–126^. Further, in contrast to single mutant mice they develop sensory premotor deficits reminiscent of the human premotor phase that manifests with a sensory loss and pain^127^ and is associated with increased plasma GlcCer species^42^. The onset of sensory deficits in *Pink1*^*−/−*^*SNCA*^A53T^ double mutant is at around 6 months of age and is progressive^127^. Using 1-year-old mice ensured the presence of PD-like pathology (mitochondrial morphology^127^, respiratory dysfunction^127^, synuclein aggregates^123^, ceramide accumulation^127^) but avoided overt serious motor deficits and suffering of the animals. Overall, *Pink1*^*−/−*^*SNCA*^A53T^ double mutant mice show many features of human PD.

Mice had free access to water and food, and they were maintained in climate-controlled rooms with a 12 h light-dark cycle. The maintenance of the breeding colony of *Pink1*^*−/−*^*SNCA*^A53T^ and observation up to onset of PD symptoms were approved by the local Ethics Committee for animal research (Darmstadt, Germany; FK1032 (12.02.205-11.02.2020) and FK1131 (17.02.2020-31.12.2023). The studies adhered to the guidelines of the Society of Laboratory Animals (GV-SOLAS) and were in line with the European and German regulations for animal research. Mice were observed for ~12 months and were euthanized to obtain tissue and plasma samples for lipid analyses before onset of overt clinical symptoms which occur in about 20 percent of mice >18 months^63,123^.

### Mice tissue collection: brain and plasma

Mice were euthanized with carbon dioxide and blood withdrawal by cardiac puncture, whereby blood was collected into K3^+^ EDTA tubes, centrifugated at 1300 × *g* for 5 min, and plasma was transferred to a fresh tube and snap frozen on dry ice or in liquid nitrogen. The brain was dissected for lipidomic and proteomic analyses. Cerebellum and olfactory bulb were removed, and the brain was cut sagittal. Left and right halves were weighed with precision scales and snap frozen on dry ice. Samples were stored at −80 °C until analysis.

### HT22 cell culture, αSyn-PFF loading, and immunofluorescence analysis

HT22 is an immortalized mouse hippocampal neuronal cell line which are derived from hippocampal neuronal HT4 cells and are widely used as an in vitro model of PD because of their high sensitivity to glutamate excitotoxicity and PD-inducing toxicants like MPP+ (1-methyl-4-phenylpyridinium) and rotenone^128,129^. They express dopamine D4 receptors^130^.

HT22 mouse immortalized hippocampal neurons (RRID:CVCL_0321) were grown in High Glucose Dulbecco’s modified eagle medium (DMEM, Gibco) containing 10% fetal bovine serum (FBS, Gibco), 2 mM L-glutamine (Gibco) and 1x penicillin/streptomycin (Sigma) at 37 °C and in 5% CO_2_ atmosphere in a humidified cell culture incubator.

For stimulation of HT22 neurons with pre-formed active aSyn fibrils (aSyn-PFF) (A53T Mutant Alpha Synuclein Active Protein, MyBioSource), sub-confluent cells were trypsinized, pelleted, and resuspended in full medium containing αSyn-PFF and seeded in T75 flasks (Greiner) to achieve a final concentration of 2 µg/ml PFF or 6 µg/ml PFF and cell density of 0.4 ×106 cells/flask. Control cells were plated in full medium without PFF.

For lipid analyses cells were incubated for 72 h and harvested by trypsinization. Cells were washed 3-times with 1× PBS, resuspended in 1 ml 1× PBS, counted and adjusted to 250,000 cells per sample, finally pelleted. The supernatant was discarded and pellets stored at −80 °C until analysis. For lipid analysis, 8 replicates were generated for each treatment.

For analysis of cell viability upon exposure with αSyn-PFF, WST-1 and LDH activity assays were used. The WST-1 assay is based on the cleavage of the water-soluble tetrazolium salt by cellular enzymes to a formazan dye, which is quantified by measuring absorbance and correlates with the number of metabolically active viable cells. Cells were seed in 96-well plates (3500 cells/well) and were cultured for 24 h or 48 h with/without 2 µg/ml αSyn-PFF. The medium was then replaced with 100 μl of fresh medium (per well), and 10 µl WST-1 solution were added to each well. After 90 min in the incubator, the absorbance was read on a multimode microplate reader (SpectraMax i3X, RRID:SCR_026346) at 450 nm, and the reference wavelength of 620 nm.

For analysis of LDH activity, 5 µl cell culture supernatant and NADH standards (0–12.5 nmol/well) were pipetted into the wells of a 96 well plate. The master reaction mix consisting of assay buffer and substrate mix was added to the wells. The absorbance was measured on a microplate reader at 450 nm every 5 min until the value of the most active sample exceeded the value of the highest standard. The absorbance was corrected with the blank, and LDH activity was calculated as described in the manufacturer’s protocol (Sigma).

For live cell immunofluorescence analyses of αSyn-PFF uptake, HT22 cells were seeded in 8-well cover glass bottom culture slides in full medium in the presence of 1 µM GlcCer24:1 (AvantiPolar Lipids #860549) or vehicle (2:1 mixture of chloroform and methanol, final dilution 1:10000) for 48 h. The chambers were coated with Poly-L-lysine (0.01%, Sigma) before seeding of 5000 cells per well. Then 2 µg/ml αSyn-PFF labeled with a fluorophore (Alexa Fluor 555 Microscale Protein Labeling Kit, Invitrogen) were added to the medium and incubated for 24 h. Live culture images were obtained on a Leica Stellaris 8 fluorescent confocal microscope (RRID:SCR_024660). To visualize the plasma membrane and the nucleus, cultures were incubated 15 min before imaging with wheat germ agglutinin-Alexa Fluor 488 reagent (WGA, Invitrogen). Hoechst 33342 was used as nuclear stain (Thermo Scientific). For analysis in FIJI ImageJ, mono-channel images were converted to 8-bit images, stacked and arranged in montages. Using the threshold default-algorithm, images were converted to binary masks which were then submitted to the particle counter for each image, and the immunofluorescent (IF) area was used for further quantitative analysis. The red-PFF IF area was normalized by the respective Hoechst IF area representing the nuclei, and the area ratio was submitted to statistical *t*-test comparisons.

### Beta-arrestin screening of G-protein activation by GlcCer24:1

PRESTO-Tango beta-arrestin assay^131^ was used to screen for the activation profile of heterologously expressed G-protein coupled receptors.

The assay uses HTLA cells which are HEK293T cells stably expressing a luciferase reporter gene and stably expressing a human β-arrestin-2 fused to Tobacco Etch Virus protease. 5000 HTLA cells were seeded in a white, transparent and poly-L-Lysin-coated 384-well plate from PerkinElmer. The cells were co-transfected after 6 h with a plasmid from the PRESTO-Tango kit (Addgene #1000000068). We used a mixture of 10 ng plasmid and 0.04 µl Lipofectamine 2000 per well performing the transfection using the protocol described by Kroeze et al.^131^. We used GFP as a transfection control and 100 µM carbachol as an assay control at the muscarinic M5 receptor. After 24 h, the medium was aspirated and replaced by 45 µl of serum-free medium. The ligand (5 µl) was then added at a final concentration of 1, 5 and 10 µM for ~24 h. Afterwards, the medium was aspirated, and the cells lysed using 50 µl of bright-Glo reagent (Promega) diluted 10X with 1x PBS. After 15 min of incubation with the lysis buffer, the luminescence (endpoint, 1500 ms integration time) was measured using a flexstation 3 device.

### Dynamic Mass Redistribution Assay (DMR)

Label-free dynamic mass redistribution enables real-time detection of refractive index alterations on biosensor-coated microplates that originate from stimulus-induced changes in the total biomass in proximity to the sensor surface. Dynamic mass redistribution monitoring was performed with a Corning EPIC BT system. 5000 HEK293 cells were seeded in a transparent and poly-L-Lysin precoated 384-well plate from Corning. The transfection was done as described for the beta arrestin screen. After 48 h, the culture medium was removed. Assay buffer was added and cells incubated in assay buffer for 2 before a 1 min baseline was recorded. Diluted lipids (final concentration 1 µM) or positive control were then added and DMR signals were acquired every 3 s for a period of 60 min. The readout is the shift of resonance wavelength over time ∆λ(t) shown as response curves. Carbachol effects on muscarinic receptors were used as positive control.

### Primary DRG neuron culture

To assess gene regulations under treatment with GlcCer24:1, primary sensory neurons were prepared from naïve adult C57BL6 mice which had free access to food and water, were kept in climate-controlled rooms with a 12 h light-dark cycle. Primary adult neuron-enriched cultures of dorsal root ganglia (DRG) of each four mice per group were prepared by dissecting DRGs of adult mice into Hank’s balanced salt solution (HBSS, Merck), followed by digestion with 5 mg/ml collagenase A (Millipore) and 1 mg/ml dispase II (Roche Diagnostics, Germany) before treatment with DNase (Sigma, 250 U per sample). Triturated cells were centrifuged through a 15% fat-free bovine serum albumin (BSA) solution. Primary sensory neurons were seeded on poly-L-lysine and laminin-coated cover slips (9–12 cover slips per mouse). Cells were cultured in serum-free Neurobasal medium (Gibco) containing 1x B27 supplement, 1x Pen/Strep, 200 ng/ml nerve growth factor and 2 mM L-glutamine at 37 °C and 5% CO_2_ and 95% humidity. Neurons were cultured in the presence of 1 µM GlcCer24:1 or vehicle (2:1 mixture of chloroform and methanol, final dilution 1:10000) and submitted to RNAseq.

### RNA sequencing in DRG neurons upon stimulation with GlcCer24:1

The day after seeding of primary DRG neurons, GlcCer24:1 was added to the medium at a final concentration of 1 µM. Control cells received the same volume of vehicle. Cells were collected two days after adding treatments. Total RNA was isolated using a single-cell RNA extraction kit (PicoPure, Thermo Fisher). Quantity of total RNA was determined using Invitrogen’s Qubit HS assay and quality was checked on an Agilent 2100 Bioanalyzer Instrument (RRID:SCR_018043). First-strand cDNA was prepared and amplified from 1 ng total RNA using SMART Seq v4 Ultra Low-Input RNA Kit for sequencing (TaKaRa) according to the manual compatible with Illumina NGS. Barcoded sequencing libraries were subsequently prepared using the low-input library preparation kit (Sv4 PLUS kit, TaKaRa). The Sv4 PLUS kit includes the first stand cDNA synthesis using SMART technology and library preparation that incorporates enzymatic fragmentation and stem-loop adapters to construct high-quality, Illumina-compatible libraries. Concentrations were determined using Invitrogen’s Qubit HS assay and fragment size was analyzed on Agilent’s 2100 Bioanalyzer on a HS DNA chip. RNA sequencing was performed of 4 biological replicates per group using an Illumina NextGen 2000 system (RRID:SCR_023614).

The sequence alignment was done with Qiagen’s CLC genomic workbench (v. 22, RRID:SCR_011853). Sequence reads were trimmed for adapter sequences and low-quality sequences using CLC genomic workbench standard settings of RNA sequencing (quality limit 0.05). Sequence reads were aligned to the reference genome mm10 provided from UCSC (GRCm38) as template, using CLC’s default setting for RNAseq. Read counts extraction and normalization (TMM) were done using CLC genomic workbench. The expression value unit is the trimmed mean of M-values, TMM (EdgeR algorithm). TMM reads were Log2 transformed.

Differential gene expression was assessed using *t*-tests and fold change using CLC genomic workbench. Genes were filtered for at least 4 valid values out of 8 samples to exclude low-expression genes. The *P* value was set at 0.05 and adjusted according to the False Discovery Rate (FDR). Hierarchical clustering with Euclidean distance metrics was used to assess gene expression patterns. Genes were ranked according to *P* and *q* value, fold change and abundance. Ranked genes were submitted to gene ontology enrichment analysis using DAVID (Database for Annotation, Visualization and Integrated Discovery^132^; RRID:SCR_001881) and ExpressAnalyst (https://www.expressanalyst.ca/) (RRID:SCR_025651)^133^. The RNAseq data have been deposited via the Gene Expression Omnibus (RRID:SCR_005012) as GEO dataset with the provisional accession number GSE262573.

### Lipidomic and metabolomic analyses (plasma, cells, supernatant)

Human blood was collected in 9 ml K3 + EDTA tubes, mouse blood in 500 µl K + EDTA tubes, centrifuged at 1300 × *g* or 1500 × *g* and plasma was transferred into 1.5 ml tubes and stored at −80 °C until further use. Cells were collected by trypsinization, washed, pelleted, resuspended in 100 µl counted and adjusted to yield 1.5x 10exp6 cells per pellet (experiment-1) or 2.5x 10exp5 cells (experiment-2). Finally, the cells were pelleted at high speed and supernatant removed with 100 µl and subsequently 20 µl pipet. The “dry” pellet was frozen at −80 °C until lipid/metabolite extraction. Fibroblasts were homogenized as explained below (targeted analyses). Lipidomic and metabolomic analysis were conducted applying the same procedure as previously described^134^. Lipidomic analysis of the human plasma samples used a slightly different procedure^135^. Targeted sphingolipid analyses were done in plasma, cell and tissue extracts essentially as described previously^42^ using liquid chromatography-electrospray ionization-tandem mass spectrometry (LC-ESI-MS/MS), according to procedures described in detail in ref. ^136^. Further protocol details for extraction and analyses of different matrices (cells, tissue, plasma) are described in the supplementary methods (Excel file).

### Statistics

Group data are presented as mean ± SD or median ± IQR as specified in the respective figure legends. Data were analyzed with SPSS 29 (RRID:SCR_016479) and GraphPad Prism 9 or 10 (RRID:SCR_002798), Origin Pro 2024 (RRID:SCR_014212), and MetaboAnalyst 5.0 (RRID:SCR_016723) (https://www.metaboanalyst.ca)^137^. Bioinformatic analysis of “omic” data (RNAseq, lipidomic, metabolomic) is explained in the respective paragraphs. Area under the curve (AUC) data of lipidomic and metabolomic analyses transformed to square root values to adjust skewed distributions. For testing the null-hypothesis that groups were identical, two groups were compared with 2-sided, unpaired Student’s *t* tests. Lipidomic and metabolomic data were submitted to 2-way analysis of variance (ANOVA) using e.g., the factors “feature” (e.g. lipid, metabolite) and ‘group’ (e.g. PD versus HC with/without stimulation). In case of significant differences, groups were mutually compared using post hoc *t*-tests according to Šidák or false discovery rate (FDR). The meaning of asterisks in figures is explained in the legends. ANOVA-simultaneous component analysis (ASCA)^138^ was used for analysis of multiple covariates on lipidomic data from patients and fibroblasts. ASCA is a combination of ANOVA and PCA plus feature extraction method for multivariate data to model two major components and their interaction. The meta-data for patients included sex, age, BMI, telomere length, and sensory loss. For multivariate analyses, data were normalized to have a common mean and variance of 1 (Z-scores = (x − x̄)/SD). Volcano plots were used to assess fold differences of lipids versus the negative logarithm (Log10) of the *t*-test *P* value according to standard procedures. Partial Least Square Discriminant analysis (PLS-DA) or canonical discrimination analysis were used to assess group membership and variable importance.

## Supplementary information

Supplementary figures and tables

Supplementary Excel file

Supplementary information

## Data Availability

All data that were analyzed for the study are presented within the manuscript or supplementary files. The source datasets supporting the conclusions of this article are available in the following repositories: RNAseq data have been deposited to NCBI's GEO repository with the accession number GSE262573. Processed lipidomic data have been deposited BioStudies (https://www.ebi.ac.uk/biostudies). The accession numbers are given below the respective results. The datasets are:Plasma lipidomic data of PD patients and healthy controls: S-BSST1880, https://www.ebi.ac.uk/biostudies/studies/S-BSST1880?key=a0dc6f53-0365-410a-96e3-d83e51a04282. Targeted sphingolipids in cells extracts of primary PD and HC fibroblasts with/without pimozide: S-BSST1881. https://www.ebi.ac.uk/biostudies/studies/S-BSST1881?key=4e8ae53f-17f0-4179-80ff-40392e418270. Untargeted lipidomics and targeted sphingolipids of brain tissue of Pink1SNCA versus wildtype mice: S-BSST1888, https://www.ebi.ac.uk/biostudies/studies/S-BSST1888?key=6230dc37-d998-4eca-bf0f-3502f7164655. Targeted and untargeted lipidomic and metabolomic analyses in HT22 cells extracts with/without preformed fibrils: S-BSST1897, https://www.ebi.ac.uk/biostudies/studies/S-BSST1897?key=d2a20a65-a88f-44a4-9654-672a72f8a9ac.
