# Supplementary material for: Elevated hexosylceramides in Parkinson’s disease cause gene upregulations in neurons mimicking responses to pathogens
Source: NPJ Parkinsons Dis. 2025 Aug 30;11:268. doi: 10.1038/s41531-025-01114-9 (PMC12398573; doi:10.1038/s41531-025-01114-9)
Supplement: Supplementary file 1 — Supplementary figures and tables [file 41531_2025_1114_MOESM1_ESM.pdf]

Supplementary Tables and Figures to

Elevated hexosylceramides in Parkinson's disease cause gene upregulations in neurons mimicking responses to pathogens

Luisa Franck[1], Lisa Hahnefeld[1,2], Lucie Valek[1], Katharina Klatt-Schreiner[1], Annett Wilken-Schmitz[1], Mohamad Wessam Alnouri[3], Sandra Trautmann[1,2], Marc-Philipp Weyer[1], Dominique Thomas[1,2], Robert Gurke[1,2], Stefan Offermanns[3], Gerd Geisslinger[1,2], Irmgard Tegeder[1]

[1]Goethe University Frankfurt, Faculty of Medicine, Institute of Clinical Pharmacology, Theodor-Stern-Kai 7, 60590 Frankfurt am Main, Germany.
[2]Fraunhofer Institute for Translational Medicine and Pharmacology ITMP and Fraunhofer Cluster of Excellence for Immune Mediated Diseases CIMD, Theodor-Stern-Kai 7, 60596 Frankfurt am Main, Germany
[3]Max Planck Institute for Heart and Lung Research, Department of Pharmacology, Bad Nauheim, Germany

Supplementary Tables

Suppl. Table 1: Demographic data for plasma lipidomic analyses

Demographic data - Samples for plasma lipidomic analyses. Modified from Mov Disord. 2020 Oct;35(10):1822-1833. doi: 10.1002/mds.28186.

Demographic data

	Healthy controls (HC)				Parkinson's Disease (PD)			
	female (n = 25)		male (n =25)		female (n = 16)		male (n = 34)	
	Mean	SD	Mean	SD	Mean	SD	Mean	SD
Age (years)	60.56	7.10	65.28	9.00	64.06	8.64	68.15	7.75
BMI	26.51	4.95	27.84	4.43	25.61	4.68	26.61	3.93
Disease Duration (years)					6.00	5.55	8.85	7.48
VAS Avg. pain	2.24	2.18	0.95	1.80	3.89	1.95	3.37	2.73

		Healthy controls (HC)						Parkinson's Disease (PD)						Counts	
		female			male			female			male			**Sum HC**	**Sum PD**
		Age Class			Age Class			Age Class			Age Class				
Medication		<= 60	61 - 70	70+	<= 60	61 - 70	70+	<= 60	61 - 70	70+	<= 60	61 - 70	70+		
Levodopa Carbidopa	no	13	9	3	9	7	9	2	0	1	2	1	4	50	10
	yes							4	5	4	6	8	13		**40**
DA agonists	no	13	9	3	9	7	9	2	2	3	1	2	6	50	16
	yes							4	3	2	6	7	10		**32**
Amantadin	no	13	9	3	9	7	9	6	4	5	7	7	11	50	40
	yes							0	1	0	0	2	5		**8**
MAOB Inhibitors	no	13	9	3	9	7	9	1	4	2	4	5	9	50	25
	yes							5	1	3	3	4	7		**23**
COMT Inhibitors	no	13	9	3	9	7	9	4	4	5	5	6	10	50	34
	yes							2	1	0	2	3	6		**14**
Anticholinergics	no	13	9	3	9	7	9	6	5	5	7	7	16	50	46
	yes							0	0	0	0	2	0		**2**
HoehnYahr	0.0	13	9	3	9	7	9	0	0	0	0	1	0	50	1
	1.0							2	2	1	0	0	2		7
	2.0							3	0	1	3	2	4		13
	2.5							0	1	2	0	1	4		8
	3.0							1	2	1	4	3	4		15
	4.0							0	0	0	1	2	3		6
Pain	no	7	3	1	7	6	6	0	0	1	4	4	4	30	13
	yes	6	6	2	2	1	3	6	5	4	4	5	13	20	37
Pain medication	no	11	6	1	7	4	4	3	3	4	6	5	9	33	30
	yes	2	3	2	2	2	5	2	2	1	2	4	8	16	19

Suppl. Table 2: Demographic data of skin biopsy donor PD patients

GR	Age	sex	Sample-Veh	Sample-PIM	HoehnYahr	Disease (years)	L-DOPA	DR-agonist	MAOB-I	COMT-I	NMDA	ACH	ApoMOR	DBS
PD	61	f	PD_veh_f_JH	PD_PIM_f_JH	1.5	1	no	no	yes	no	no	no	no	no
PD	55	f	PD_veh_f_KP	PD_PIM_f_KP	1		no	yes	no	no	no	no	no	no
PD	64	m	PD_veh_m_FB	PD_PIM_m_FB	3	26	yes	yes	no	yes	yes	no	yes	no
PD	75	m	PD_veh_m_HJI	PD_PIM_m_HJI	3	7	yes	no	no	no	no	no	no	no
PD	59	f	PD_veh_f_HS	PD_PIM_f_HS	4	11	yes	no	no	yes	no	no	no	yes
PD	56	f	PD_veh_f_EM	PD_PIM_f_EM	1	3	no	yes	yes	no	no	no	no	no
PD	78	m	PD_veh_m_OH	PD_PIM_m_OH	3	21	yes	yes	no	yes	no	no	no	yes
PD	79	m	PD_veh_m_GRM	PD_PIM_m_GRM	3	16	yes	no	yes	yes	no	no	no	no
PD	72	m	PD_veh_m_GS	PD_PIM_m_GS	2	2	no	yes	no	no	no	yes	no	no
PD	84	m	PD_veh_m_MG	PD_PIM_m_MG	2	4	yes	yes	no	no	no	no	no	no
PD	58	m	PD_veh_m_TSch	PD_PIM_m_TSch	3	10	yes	no	no	yes	no	no	no	yes
PD	74	m	PD_veh_m_RK	PD_PIM_m_RK		3	no	yes	no	no	no	no	no	no
PD	65	m	PD_veh_m_HJB	PD_PIM_m_HJB			yes	no	no	no	no	yes	no	no

Supplementary Figures

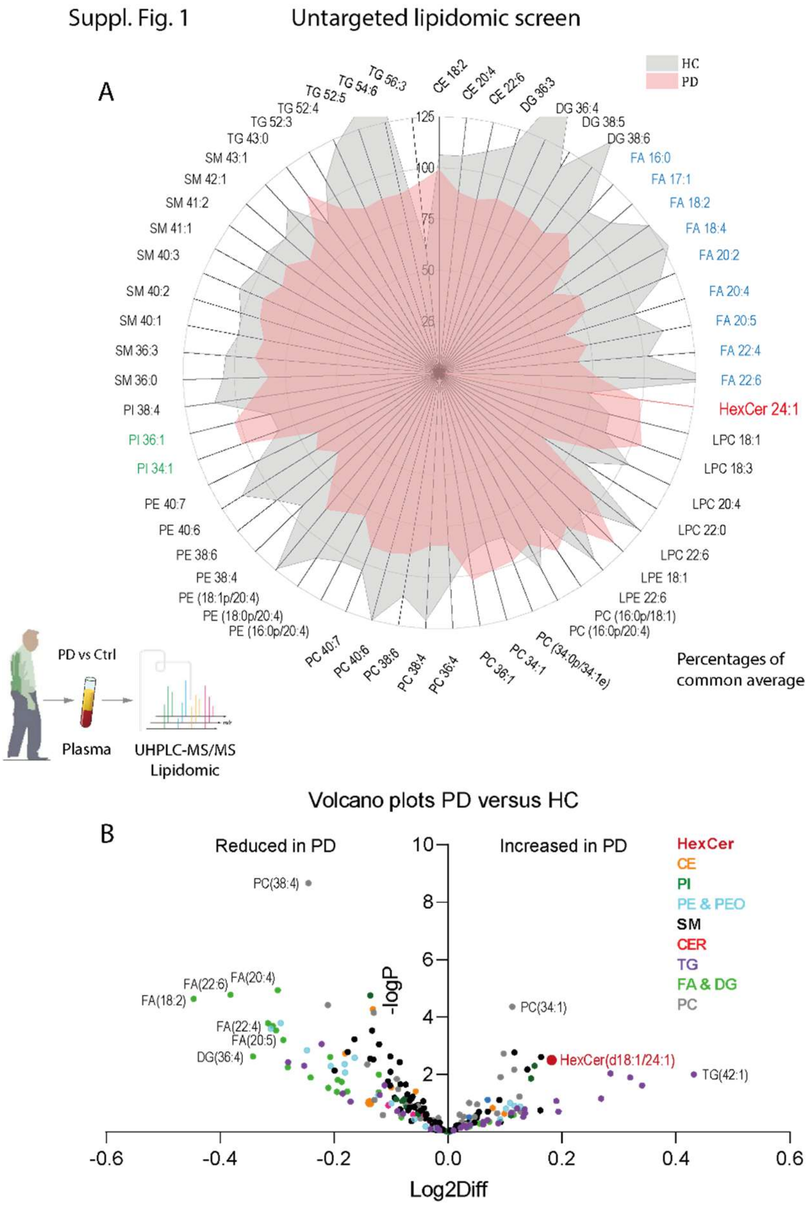

Supplementary Figure S1

Plasma lipidomic analyses in PD patients versus healthy controls (HC)

Parkinson’s Disease (PD) patients (n = 16 female, 34 male) and healthy controls (HC) (n = 25 female, 25 male) were age matched 60-70+ years old at the time of blood sampling. Demographic details as in {Klatt-Schreiner, 2020 #415}.

A: The radial plots shows top down- and upregulated lipids in PD versus HC. Mass spectrometry area under the curve (AUC) were divided by the AUC of the internal standard (IS) (AUC/IS). Because lipid abundance varies over several orders of magnitude, AUC/IS values were transformed into percentages versus a common average for each lipid. The mean percentages per group are presented in the radial plot. For clarity, the plot shows no error bars.

B: Volcano plots show the Log2(fold change) on the x-axis versus the negative Log10 of the P-value of t-tests on the Y-axis. The lipid classes are colour coded.

Abbreviations: CAR, carnitines; CER, ceramides; CE, cholesterol ester; DG, diglycerides; FA, fatty acids; HexCer, hexosylceramides; LPC, lysophosphatidylcholines; LPE, lysophosphatidylethanolamines; LPG, lysophosphatidylglycerols; LPI, lysophosphatidylinositols; PC, phosphatidylcholines; PE, phosphatidylethanolamines; PD, phosphatidylglycerols; PI, phosphatidylinositols; SM, sphingomyelins; ST, sterols; TG, triglycerides; UbiQ, ubiquitin; –O ether bound.

Suppl. Fig. 2

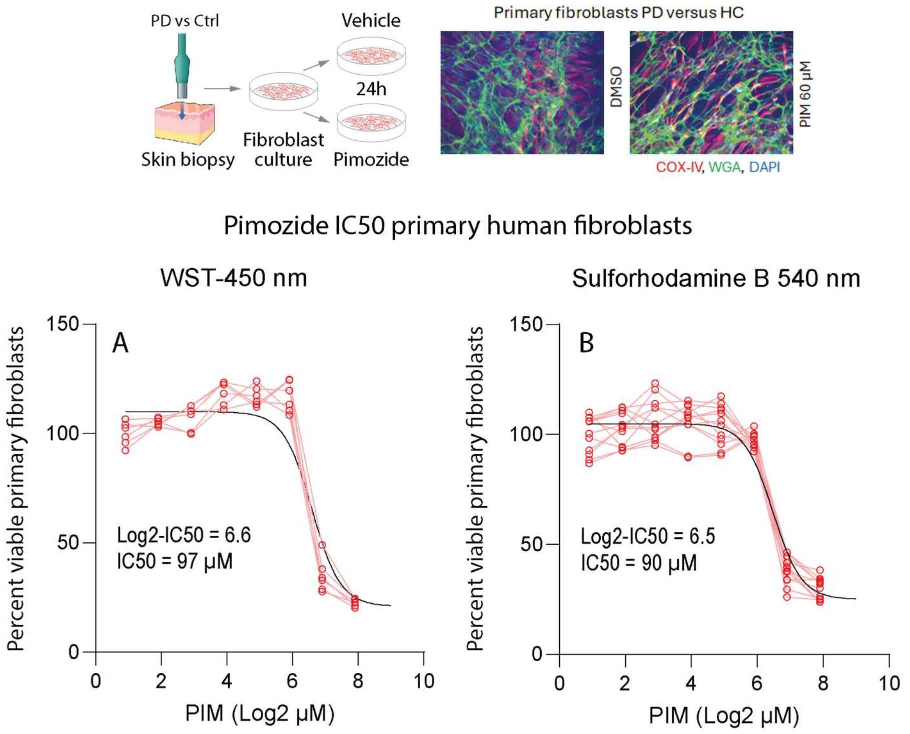

Supplementary Figure S2

Pimozide IC50 in primary human fibroblasts

In a 96-well plate, PHF cultures were treated for 24h with pimozide at increasing log2-scaled eight concentrations in rows A-H. Viability was assessed at 24h using WST reagent and sulforhodamine B (SRB) assays. WST absorbance and SRB absorbance were transformed into percentage survival as compared to the average of vehicle treated cultures and plotted on the Y-axis versus the Log2 pimozide concentration on the X-axis. Each scatter represents one culture. The red lines connect cultures in one column of the 96-well plate. Data were analysed using as standard sigmoidal inhibitory Emax model. The fit line is shown in black. The IC50 of pimozide was 90-100 µM.

Suppl. Fig. 3 Lipidomic analysis brain tissue top 70

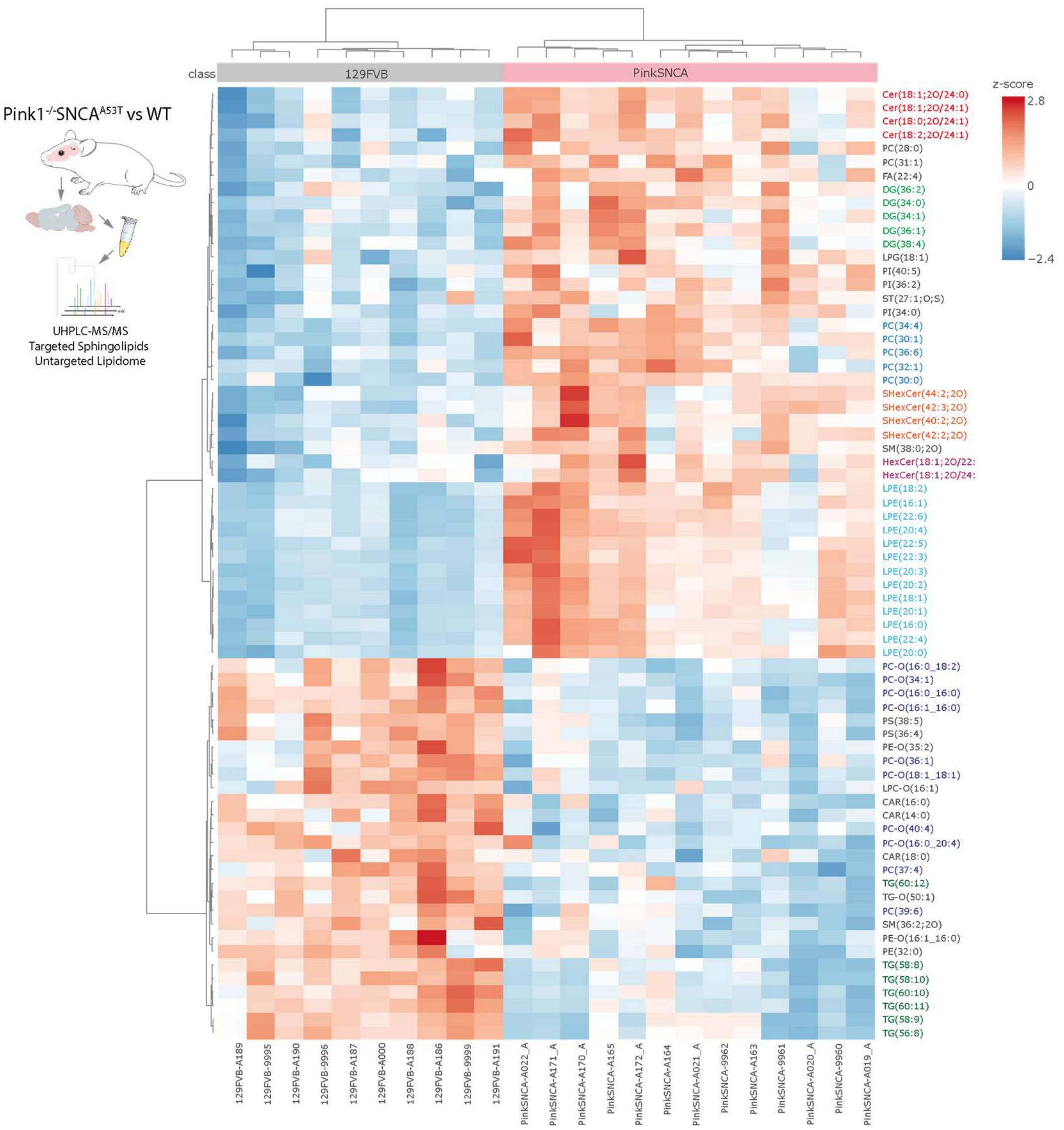

Supplementary Figure S3

Lipidomic UHPLC-MS/MS analysis of mouse brain in Pink1$^{-/-}$SNCA^{A53T} mice and WT controls

Heatmap of top 70 regulated lipids in Pink1$^{-/-}$SNCA^{A53T} mouse brain versus wildtype controls brain (Sv129-FVB). Brainstem, cerebellum and olfactory bulb were removed and the brain cut saggital. One half was used for UHPLC-MS/MS lipidomic analysis. Lipid expression values (AUC divided by the AUC of the internal standard; AUC /IS) were square root transformed and auto-scaled to have a common mean and variance of 1 [Z score = (x- x̄)/SD]. Lipids were clustered according to Euclidean distance metrics using the Ward method. For easier evaluation, lipid species belonging to the same class are depicted in colour. Columns clustered in two clusters according to genotypes. The bottom line shows the IDs of individual mice (n = 10 Sv129FVB controls, n = 13 Pink1$^{-/-}$SNCA ^{A53T} double mutant PD mice).

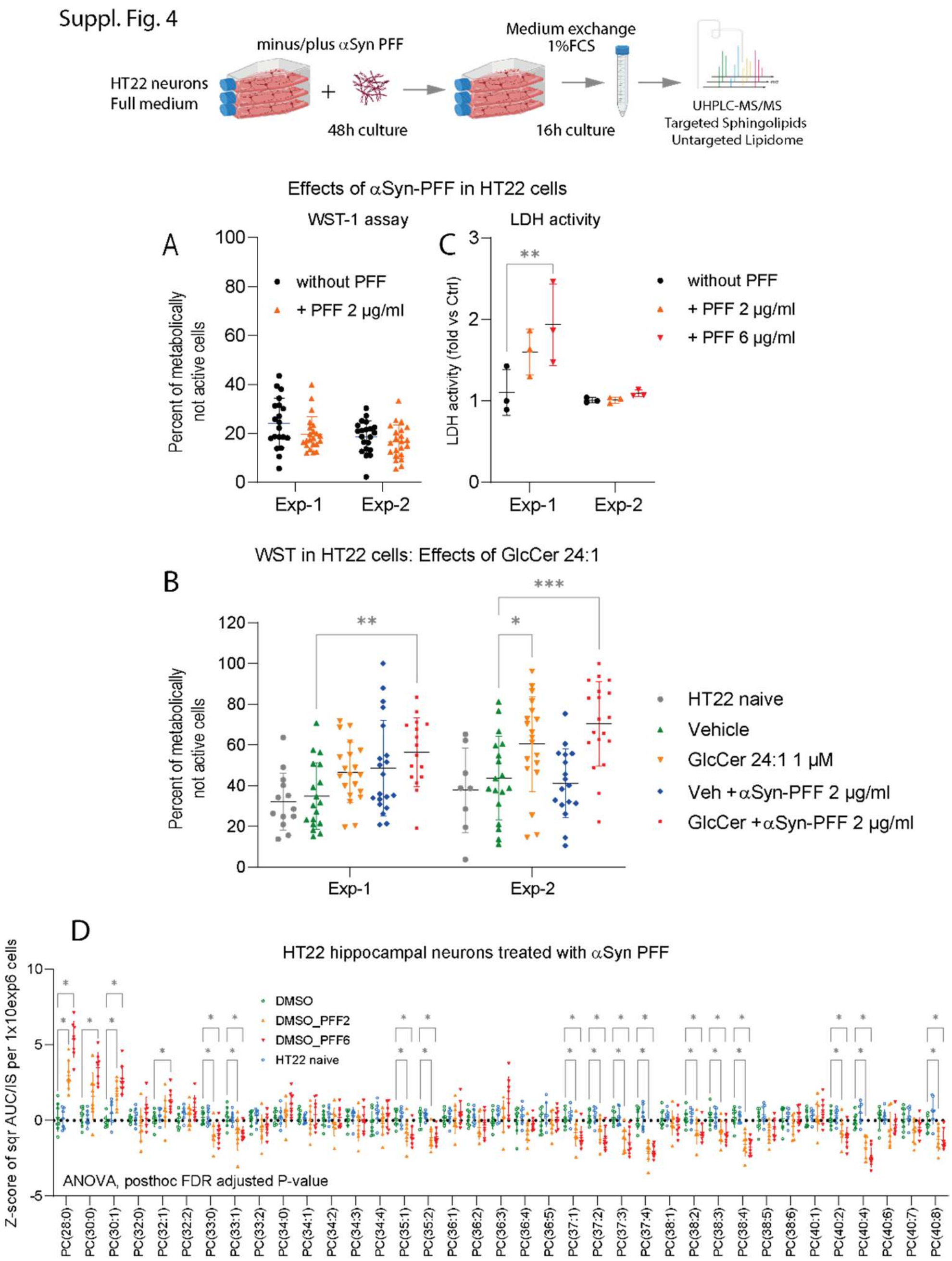

Supplementary Figure S4

HT22 mouse hippocampal neurons viability treated with alpha synuclein preformed fibrils (PFF)

A, B: HT22 were seeded into 96-well plates in the presence or absence of αSyn-PFF and GlcCer24:1, and the WST-1 (water soluble tetrazolium) assay was performed 48h after seeding. The decrease of absorbance indicates a reduction of metabolically active viable cells. The data in A and B are each from two consecutive independent experiments. Scatters are replicates. Errors bars are SD. αSyn-PFF per se had no impact on cell viability, but in combination with GlcCer 24:1 the fraction of non-viable cells was increased. **C:** LDH activity in HT22 cell culture supernatants in the presence or absence of αSyn-PFF. LDH activity was measured with a colorimetric enzyme activity assay. ANOVA for group comparisons, asterisks indicate statistically significant differences, *P<0.05, **P<0.01, ***P<0.001.

D: Treatment of HT22 with αSyn-PFF caused a shift of phosphatidylcholine species from long-chain PC to short-chain PC. Z-scores of AUC/IS values were submitted to ANOVA and subsequent posthoc t-tests using an FDR adjustment of alpha. Asterisks show significant discoveries.

Suppl. Fig. 5

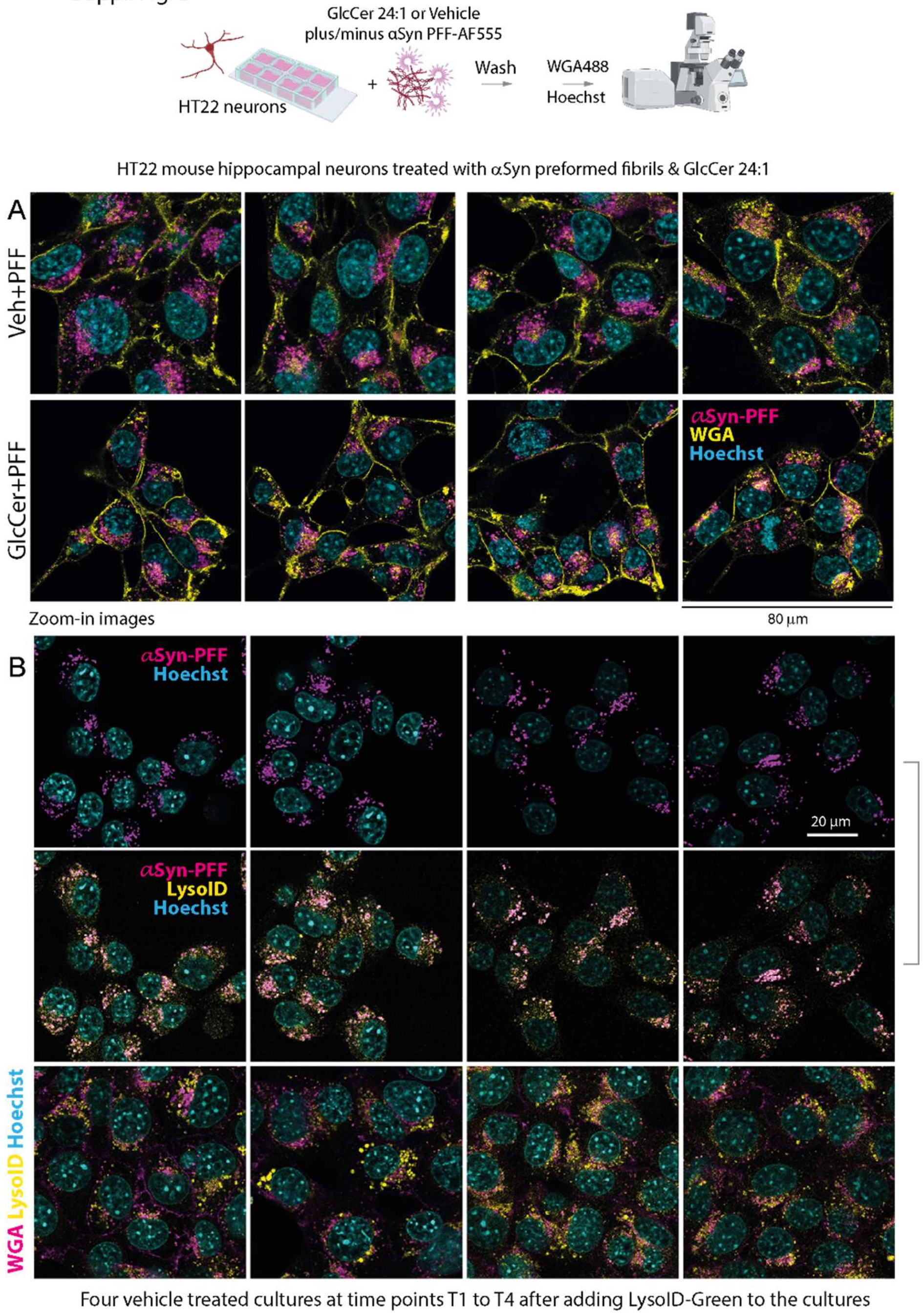

Supplementary Figure S5

Cellular uptake of preformed alpha synuclein fibrils (αSyn-PFF) in HT22 cells

A: Corresponding to figure 5 (main body) the images show zoom-in images of the uptake of AF555-labeled αSyn preformed fibrils in HT22 mouse hippocampal neurons (2 µg/ml αSyn-PFF). The images are each 4 examples taken at early and late time points in two independent consecutive replicate experiments. Pretreatment with GlcCer24:1 (1 µM) had no significant impact on the PFF-AF555 fluorescence uptake in HT22 cells.

B: To show the intracellular localization of αSyn-PFF (pink) lysosomes are counterstained with LysoID®-Green (yellow). Hoechst 33342 (blue) was used as nuclear counterstain. The upper row shows αSyn-PFF and Hoechst alone, the middle row in combination with LysoID. αSyn-PFF and LysoID overlap. The bottom row shows WGA-AF594 in combination with LysoID® and Hoechst to reveal the translocation of WGA from plasma membrane to endolysosomes over time.

Suppl. Figure 6

β-Arrestin assay of candidate GPCR in HEK293 cells

Fold change versus average of vehicle (0 µM)

GlcCer18:1	ADORA1				ADRA2B				CHRM3				GPR63				GPR75			
0 µM	0.81	1.29	1.14	0.76	0.95	0.85	1.38	0.82	0.91	1.27	0.89	0.93	1	0.56	1.54	0.91	0.87	0.79	1.1	1.24
1 µM	2.4	1.77	2.57	0.84	0.69	0.82	0.6	0.93	0.69	0.65	1.32	2.37	0.89	0.8	1.95	1.35	1.11	0.99	0.53	0.61
5 µM	1.05	1.88	0.73	1.49	1.73	0.98	0.53	0.89	0.67	3.76	0.89	0.53	1.92	0.83	1.37	0.96	0.69	0.95	1.36	1.22
10 µM	0.97	1.4	1.66	0.9	0.73	1.18	1.04	0.78	0.89	1.37	0.67	0.62	0.97	0.98	1.01	0.91	0.94	0.74	1.36	0.91

GlcCer18:1	HRH1				HRH4				LPAR1				NPBW2				OXGR1				P2RY1			
0 µM	0.65	0.85	1.7	0.8	1.09	1.24	0.84	0.84	0.86	0.51	1.58	1.05	0.83	0.94	1.21	1.02	0.68	0.5	1.35	1.47	0.35	0.33	1.71	1.61
1 µM	0.65	1	2.25	1.22	2.06	1.22	0.53	1.37	1.33	0.99	1.41	0.76	1.07	1.44	6.61	2.65	0.88	0.96	1.26	1.31	1.65	1.62	1.02	1.88
5 µM	0.67	0.61	1.99	2.03	2.46	1.24	0.79	2.26	0.76	0.34	2.08	0.75	1.17	1.74	2.14	1.73	0.92	0.65	1.53	1.06	0.49	0.32	1.37	1.8
10 µM	1.45	0.48	0.77	0.93	4.13	1.76	1.25	1.13	1.95	0.88	0.79	0.29	2.46	1.25	1.9	2.03	1.19	0.47	1.2	0.99	1.97	1.76	0.42	0.43

Fold change versus average of vehicle (0 µM)

GlcCer24:1	GAL1				GRM5				MRGPRF				OPRL1				SSTR4				P2RY1			
0 µM	0.89	1.3	0.74	1.07	1.19	0.82	1.1	0.89	0.69	1.61	0.9	0.8	0.63	0.73	1	1.64	0.63	1.13	1.01	1.24	0.77	0.23	1.29	1.71
1µM	0.35	0.62	0.25	0.61	0.42	0.52	0.41	0.52	0.83	0.8	0.9	1.11	1.74	1.27	1.15	1.92	1.03	0.78	0.71	1.05	0.33	0.49	4.73	0.75
5µM	0.39	0.67	0.37	1.19	0.7	0.55	0.61	0.94	1.54	1.49	0.76	1.37	1.71	2.22	1.27	1.64	0.55	0.95	1.8	1.1	0.24	1.2	4.5	1.98
10µM	0.67	1	0.75	1.02	0.72	1.19	1.41	2.1	1.75	1.63	2.41	2.13	2.81	2.83	3.08	3.96	1.38	1.83	1.19	2.58	4.36	0.25	1.29	1.94

	Positive control - Muscarin receptor stimulation carbachol												Positive control - Muscarin receptor stimulation carbachol											
	CHRM1				CHRM5				Untransfected				CHRM1				CHRM5				Untransfected			
0 µM	0.84	0.84	0.72	1.59	0.92	1.02	1.13	0.92	0.92	0.87	1.32	0.89	0.7	0.51	2.07	0.72	0.71	0.45	1.03	1.82	0.96	1.64	0.72	0.68
1µM	Experiment GlcCer18:1								0.56	0.94	0.78	0.99	Experiment GlcCer24:1								0.32	1.28	0.56	1
5µM									0.73	1.2	0.8	1.48									0.96	0.8	1.72	1.12
10µM									1.6	1.04	0.78	1.22									1.6	3.6	2.04	2.56
100µM	43.6	35.6	67.3	47.8	5.09	3.92	4.59	5.24					51.8	52.7	44.8	37.6	6.16	6.81	6.71	6.97				

Supplementary Figure S6

Replicates analyses of beta arrestin-based GPCR screening assay

GPCR activity was assessed in a heterologous expression model in COS cells stimulated with GlcCer 18:1 versus vehicle or GlcCer 24:1 versus vehicle at 1, 5 and 10 µM. The heatmap shows replicate analyses of GPCR candidates which were selected from an initial screen (330 GPCRs, 8 neg and 4x2 pos controls).
